# Effects of different black soldier fly larvae products on slow-growing broiler performance and carcass characteristics

**DOI:** 10.1016/j.psj.2024.103481

**Published:** 2024-01-19

**Authors:** Anna Dörper, Henrieke M. Berman, Gerrit Gort, Jan van Harn, Marcel Dicke, Teun Veldkamp

**Affiliations:** ⁎ Laboratory of Entomology, Wageningen University & Research, Wageningen, 6700AA, the Netherlands; † Animal Nutrition Group, Wageningen University & Research, Wageningen, 6700AH, the Netherlands; ‡ Biometris, Wageningen University & Research, Wageningen, 6700AA, the Netherlands; § Wageningen Livestock Research, Wageningen University & Research, Wageningen, 6700AH, the Netherlands

**Keywords:** Broiler nutrition, Performance, Carcass yield, Insects as feed, *Hermetia illucens*

## Abstract

Black soldier fly (**BSF**) larvae have gained significant attention as ingredients for poultry feed to improve value chain circularity and sustainability. Black soldier fly larvae contain bioactive compounds which can potentially improve broiler health and thereby performance. However, the functionality of bioactive compounds likely depends on how larvae are processed prior to feeding and to which extent larvae products are included in the diet. This may explain the variable results reported in literature on broiler performance and carcass characteristics when feeding them different types of BSF larvae products at different inclusion levels. Therefore, the present research aimed to investigate the effects of different BSF larvae products and inclusion levels in diets on performance and carcass characteristics of slow-growing broilers. The experiment started with 1,728 one-day-old slow-growing male broilers (Hubbard JA757). Nine dietary treatments were used, each replicated eight times. One group of broilers was given a control diet. The following BSF larvae products were investigated: live larvae, a combination of BSF larvae meal and oil mimicking the nutritional composition of the live larvae, and BSF larvae meal and oil separately. All insect products were tested at two inclusion levels. All diet programs were nutritionally comparable (isoenergetic and based on balanced levels of digestible essential amino acids). During the 7-wk trial, several performance parameters and carcass characteristics were measured. The results show that comparable or better broiler performance was achieved with the inclusion of BSF larvae products in the diets compared to the control. Based on the feed conversion ratio (**FCR**), the unprocessed larvae product and the highest inclusion level led to the most favorable results. Carcass characteristics remained unchanged when BSF larvae products were used in the diets compared to the control group, indicating favorable production output. The BSF larvae products investigated seem suitable feed ingredients for broilers at the current levels tested, generating performance benefits.

## INTRODUCTION

Within the last decades insect products have gained increasing attention as a new ingredient for livestock feed (Van Huis et al., 2021). However, rather than being a newly discovered feedstuff, insects celebrate a comeback in poultry diets. The red jungle fowl, the primary ancestors of modern chicken living in the south Asian forests, consumes considerable amounts of insects to cover their nutritional needs (Collias and Saichuae, 1967; Klasing, 2005). Unlike wild chickens which rely on live insects and their remains, today's insect production offers various products made of insects with additional functional and nutritional properties, ranging from live larvae (Veldkamp and Van Niekerk, 2019), dried whole larvae (Bejaei and Cheng, 2020) to various nondefatted (Maurer et al., 2015), partially defatted (Maurer et al., 2015; Schiavone et al., 2017) or highly defatted insect meals (Schiavone et al., 2017) and insect oil (Kim et al., 2020). Most research aims to replace soybean meal, fishmeal or soybean oil (Dörper et al., 2021). The main drivers for the transition towards insects in feed are to increase sustainability and value chain circularity (Dicke, 2018; Barragán-Fonseca et al., 2020; Saatkamp et al., 2022).

In this context, black soldier fly (**BSF**) larvae have been highlighted as a promising insect species to be used in livestock feed (Barragan-Fonseca et al., 2017; van Huis et al., 2020; Dörper et al., 2021). To highlight a few benefits, the production of BSF larvae fed with residual streams has a lower environmental impact requiring less land and energy per kg protein compared to conventional protein sources (Bosch et al., 2019). Moreover, the larvae contain large quantities of protein and fat with amino acid and fatty acid profiles favorable for poultry nutrition (Veldkamp and Bosch, 2015). Further incentives to use the larvae in feed are based on poultry health-related benefits.

Insect products have been found to alleviate health-related issues associated with poultry rearing. It is often mentioned that insect products contain bioactive components such as lauric acid, antimicrobial peptides, and chitin which may improve broiler health (Gasco et al., 2018). However, this depends on whether unprocessed or processed insects are used and also on the inclusion level of insect products in the diet. While unprocessed insects still contain the entirety of those components, further processing, such as drying or separation of fat and protein, does likely not only change nutritional properties but also structure, abundance, and functionality of these components of the insect product (Veldkamp et al., 2021; Hurtado-Ribeira et al., 2023; Son et al., 2023). Animal health and performance are closely related. This may explain why results on broiler performance and meat yield differ tremendously when feeding different types of BSF larvae products and inclusion levels in the literature (Dörper et al., 2021). Studies reported positive, neutral, and negative effects on performance and meat yield in poultry (Dabbou et al., 2018; Velten et al., 2018; Schiavone et al., 2019; Attivi et al., 2020; Ipema et al., 2020b; Bellezza Oddon et al., 2021; Heuel et al., 2022; de Jong et al., 2021). To the best of our knowledge there is no literature available which directly compares the potential of live larvae vs. processed larvae within 1 study.

Furthermore, most literature focused on fast-growing broilers, while information about feeding insect products to slow-growing broilers is lacking. Slow-growing broilers have a slower growth rate than fast-growing broilers and therefore reach their slaughter weight at a later age. Under the Dutch Better Life certification scheme, broilers, such as of the breed Hubbard JA 757, count as slow growing with a growth rate less than 45 g/d (Stadig, 2019; Dierenbescherming, 2023). In comparison, fast-growing broilers, such as Ross 308, are slaughtered at an age of 35 to 42 d, resulting in a growth rate of 60 to 65 g/d (Stadig, 2019). The use of slow-growing broilers in Dutch production systems has become more popular over the past years. A recent report published by Avined highlights this transition (Avined, 2023). In the report the ratio between slow and fast growing broilers on farms is expressed as % animal days. The term animal days reflects the cumulative number of broilers present on Dutch farms each day over the course of a year. While in 2016 slow-growing broilers accounted for 5% animal d and fast-growing broilers for 95%, this ratio shifted to slow-growing broilers accounting for 48% animal d and fast-growing broilers accounting for 52% (Avined, 2023). Therefore, research should also focus on slow-growing broilers to support those production systems.

Based on the identified knowledge gaps, the present research investigated the effect of different BSF larvae products and inclusion levels in boiler diets on performance and carcass characteristics of slow-growing broilers. Black soldier fly larvae products tested were live larvae, a combination of BSF larvae meal and oil mimicking the nutritional composition of the live larvae, and BSF larvae meal and oil separately. All products were tested at two inclusion levels and diets. It was hypothesized that the insect products can be used as a replacement of conventional protein and fat sources in broiler diets. Effects on performance and carcass characteristics were expected to improve, with the greatest effects expected in the groups fed unprocessed live BSF larvae in the diet and greater effects were expected for a high inclusion level compared to a low inclusion level of insect products in the diets.

## MATERIALS AND METHODS

### Ethical Approval

The experiment was conducted between September and November 2021 in accordance with the Dutch legislation and regulations for animal experiments. The project was approved by the Central Authority for Scientific Procedures on Animals (CCD) and the experimental protocol approved by the Animal welfare body of Wageningen University, with application number AVD40100202010104.

### Study Design

In the current study, nine experimental diet programs differing in main protein and fat sources were used. A broiler diet program, designed based on programs commercially available, was given to the control group. In two diet programs live larvae were fed besides a complement diet aiming to replace 5% and 10% of the daily dry matter feed intake (DMFI) in the form of live insect larvae (L-low and L-high, respectively). The chosen inclusion levels were based on previous research in which fast-growing broilers were provided with live black soldier fly larvae (Ipema et al., 2020a; Ipema et al., 2020b). In diet programs containing BSF larvae meal and oil (MO-low and MO-high), the two products were mixed in a 2:1 ratio, to mimic the nutritional composition of the live larvae used in the diets L-low and L-high. Other diet programs only contained BSF larvae meal (M-low and M-high) or BSF larvae oil (O-low and O-high) in the same quantities as for the diets MO-low and MO-high.

### Diets

#### Formulation

BSF larvae products were obtained from Protix BV (Dongen, the Netherlands). All experimental diets were produced at Research Diet Services B.V. (Wijk bij Duurstede, the Netherlands). A three-phase diet program was used: starter (d 0–14, 2.5 mm pellet size), grower (d 15–27, 3.2 mm pellet size), and finisher diet (d 28–51, 3.2 mm pellet size). The feeds were formulated to meet the nutritional requirements of the broilers (P. van Boekholt, pers. com.). For rations containing live larvae, the daily larvae portion was prepared to replace 5% or 10% of the expected daily dry matter feed intake (P. van Boekholt, pers. com.), while the remaining 95% or 90% of the expected daily dry matter feed intake was satisfied by pelleted feed with the nutritional composition formulated to complement the nutrients of the live larvae. The diets were formulated according to standard northern European diets based on corn, wheat, and soybean meal. Within a feeding phase, the diets were iso-energetic, and based on balanced levels of digestible essential amino acids (lysine, methionine + cysteine, threonine, tryptophan, arginine, isoleucine, and valine). Values for the apparent ileal digestibility coefficients of amino acids, the apparent digestibility coefficients of the total tract of nutrients and the apparent metabolizable energy of BSF larvae meal were derived from Schiavone et al. (2017). Due to a lack of available information, it was assumed, as recommended by the producer, that the metabolizable energy value of BSF larvae oil for broilers is equal to that of coconut oil (CVB, 2021). The ingredient composition (Table 1, Table 2, and Table 3) and nutrient contents (Table S 1, Table S 2, and Table S 3) of the pelleted feed was adjusted per feeding phase and treatment. A homogenized sample of the main diet ingredients (wheat, corn, soybean meal, sunflower meal, BSF larvae meal, soybean oil, palm oil, BSF larvae oil) was collected prior to the diet production for nutritional analysis (Tables 4, 5, 6, Tables S 4 and S 5). Based on the ingredient analysis the experimental diets were formulated. After feed production, a sample of each pelleted feed per feeding phase was collected and nutritionally analyzed (Tables S 6–Table S 14). It should be noted that broilers of the treatments with live larvae were expected to meet their daily dry matter feed intake and nutritional requirements by consuming 95 or 90% pelleted feed and 5 or 10% live larvae. All ingredient and diet analyses were performed by LUFA-ITL GmbH (AGROLAB Group, Kiel, Germany). Pelleted feed and water were provided *ad libitum* throughout the experiment.

**Table 1:** Ingredients (% w/w as fed) of pelleted starter feed (d 0–14) of all experimental treatments.

Ingredients	Control	L-low	MO-low	M-low	O-low	L-high	MO-high	M-high	O-high
Wheat	15.9	17.8	16.9	16.8	15.7	19.0	17.0	17.3	15.5
Corn	45.0	47.5	45.0	45.0	45.0	50.2	45.0	45.0	45.0
Soybean meal	25.7	23.2	22.0	22.1	25.7	21.3	19.1	19.0	25.7
Sunflower meal	3.00	3.16	3.00	3.00	3.00	3.35	3.00	3.00	3.00
Black soldier fly larvae meal			3.49	3.49			6.99	6.99	
Soy oil	5.00	2.63	2.49	4.05	3.49	0.11	0.10	3.16	1.95
Black soldier fly larvae oil			1.67		1.67		3.34		3.34
Limestone	1.47	1.49	1.41	1.41	1.46	1.49	1.34	1.34	1.46
Monocalcium phosphate	1.45	1.57	1.49	1.49	1.45	1.71	1.53	1.52	1.45
Vitamin & mineral premix	0.50	0.53	0.50	0.50	0.50	0.56	0.50	0.50	0.50
Sodium bicarbonate	0.46	0.50	0.47	0.47	0.45	0.52	0.47	0.48	0.45
Salt	0.07	0.04	0.04	0.04	0.07	0.03	0.03	0.03	0.07
L-Lysine	0.52	0.57	0.54	0.54	0.52	0.60	0.54	0.54	0.52
DL-Methionine	0.39	0.43	0.41	0.41	0.39	0.47	0.42	0.42	0.39
L-Threonine	0.21	0.23	0.22	0.22	0.21	0.23	0.21	0.21	0.21
L-Arginine	0.16	0.22	0.21	0.20	0.16	0.27	0.24	0.24	0.16
L-Valine	0.16	0.15	0.14	0.14	0.16	0.12	0.11	0.11	0.16
L-Isoleucine	0.08	0.09	0.09	0.09	0.08	0.10	0.09	0.09	0.08
Total	100.00	100.00	100.00	100.00	100.00	100.00	100.00	100.00	100.00

Control = Conventional corn-wheat based broiler diet.

L-low = 5% of the daily dry matter feed intake is replaced by live black soldier fly larvae.

MO-low = 5% of the daily dry matter feed intake is replaced by a mix of black soldier fly larvae meal & oil.

M-low = the same amount of black soldier fly larvae meal is used as in diet MO-low.

O-low = the same amount of black soldier fly larvae oil is used as in diet MO-low.

L-high = 10% of the daily dry matter feed intake is replaced by live black soldier fly larvae.

MO-high = 10% of the daily dry matter feed intake is replaced by a mix of black soldier fly larvae meal & oil.

M-high = the same amount of black soldier fly larvae meal is used as in diet MO-high.

O-high = the same amount of black soldier fly larvae oil is used as in diet MO-high.

**Table 2:** Ingredients (% w/w as fed) of pelleted grower feed (d 15–27) of all experimental treatments.

Ingredients	Control	L-low	MO-low	M-low	O-low	L-high	MO-high	M-high	O-high
Wheat	34.4	37.2	35.3	35.3	34.4	40.2	36.0	36.1	34.4
Corn	27.5	29.0	27.5	27.5	27.5	30.7	27.5	27.5	27.5
Soybean meal	21.3	18.9	17.9	17.9	21.3	16.3	14.6	14.6	21.3
Sunflower meal	4.00	4.22	4.00	4.00	4.00	4.46	4.00	4.00	4.00
Black soldier fly larvae meal			3.49	3.49			6.99	6.99	
Palm oil	5.48	2.98	2.83	4.34	3.98	1.12	1.00	3.20	2.47
Soy oil	2.28	2.37	2.25	2.40	2.12	1.64	1.47	2.52	1.97
Black soldier fly larvae oil			1.67		1.67		3.34		3.34
Limestone	1.37	1.38	1.31	1.31	1.37	1.39	1.25	1.25	1.37
Monocalcium phosphate	1.32	1.43	1.36	1.36	1.32	1.56	1.40	1.40	1.32
Vitamin & mineral premix	0.50	0.53	0.50	0.50	0.50	0.56	0.50	0.50	0.50
Sodium bicarbonate	0.42	0.46	0.44	0.44	0.43	0.50	0.45	0.45	0.43
Salt	0.07	0.05	0.05	0.05	0.07	0.03	0.03	0.03	0.07
L-Lysine	0.46	0.50	0.47	0.47	0.46	0.54	0.48	0.48	0.46
DL-Methionine	0.35	0.38	0.36	0.36	0.35	0.41	0.37	0.37	0.35
L-Threonine	0.20	0.21	0.20	0.20	0.20	0.22	0.20	0.20	0.20
L-Arginine	0.14	0.19	0.18	0.18	0.14	0.25	0.22	0.22	0.14
L-Valine	0.14	0.12	0.11	0.11	0.14	0.09	0.08	0.08	0.14
L-Isoleucine	0.08	0.08	0.08	0.08	0.08	0.09	0.08	0.08	0.08
L-Tryptophan		0.01				0.01			
Total	100.00	100.00	100.00	100.00	100.00	100.00	100.00	100.00	100.00

Control = Conventional corn-wheat based broiler diet.

L-low = 5% of the daily dry matter feed intake is replaced by live black soldier fly larvae.

MO-low = 5% of the daily dry matter feed intake is replaced by a mix of black soldier fly larvae meal and oil.

M-low = the same amount of black soldier fly larvae meal is used as in diet MO-low.

O-low = the same amount of black soldier fly larvae oil is used as in diet MO-low.

L-high = 10% of the daily dry matter feed intake is replaced by live black soldier fly larvae.

MO-high = 10% of the daily dry matter feed intake is replaced by a mix of black soldier fly larvae meal and oil.

M-high = the same amount of black soldier fly larvae meal is used as in diet MO-high.

O-high = the same amount of black soldier fly larvae oil is used as in diet MO-high.

**Table 3:** Ingredients (% w/w as fed) of pelleted finisher feed (d 28–51) of all experimental treatments.

Ingredient	Control	L-low	MO-low	M-low	O-low	L-high	MO-high	M-high	O-high
Wheat	37.1	41.3	39.1	41.5	41.2	43.8	39.3	39.4	38.9
Corn	24.5	25.8	24.5	24.5	24.5	27.3	24.5	24.5	24.5
Soybean meal	20.4	16.7	15.9	13.7	16.4	14.7	13.2	13.2	18.6
Sunflower meal	5.00	5.27	5.00	5.00	5.00	5.58	5.00	5.00	5.00
Black soldier fly larvae meal			3.49	3.49			6.99	6.99	
Palm oil	6.91	4.22	4.00	5.00	4.50	1.67	1.50	4.50	3.50
Soy oil	2.05	2.18	2.07	2.32	2.08	2.24	2.01	2.32	1.82
Black soldier fly larvae oil			1.67		1.67		3.34		3.34
Limestone	1.16	1.16	1.10	1.11	1.17	1.16	1.04	1.04	1.17
Monocalcium phosphate	1.03	1.14	1.08	1.09	1.06	1.24	1.11	1.11	1.04
Vitamin & mineral premix	0.50	0.53	0.50	0.50	0.50	0.56	0.50	0.50	0.50
Sodium bicarbonate	0.37	0.42	0.40	0.44	0.44	0.46	0.41	0.40	0.40
Salt	0.11	0.07	0.07	0.05	0.06	0.07	0.06	0.06	0.09
L-Lysine	0.35	0.41	0.39	0.46	0.47	0.42	0.38	0.38	0.40
DL-Methionine	0.29	0.33	0.31	0.33	0.32	0.35	0.31	0.32	0.30
L-Threonine	0.14	0.17	0.16	0.19	0.20	0.17	0.15	0.15	0.17
L-Arginine	0.05	0.13	0.12	0.18	0.16	0.16	0.14	0.14	0.10
L-Valine	0.06	0.07	0.07	0.09	0.13	0.02	0.02	0.02	0.09
L-Isoleucine	0.03	0.05	0.05	0.09	0.10	0.04	0.04	0.04	0.06
L-Tryptophan		0.01	0.01	0.02	0.02				0.01
Total	100.00	100.00	100.00	100.00	100.00	100.00	100.00	100.00	100.00

Control = Conventional corn-wheat based broiler diet.

L-low = 5% of the daily dry matter feed intake is replaced by live black soldier fly larvae.

MO-low = 5% of the daily dry matter feed intake is replaced by a mix of black soldier fly larvae meal and oil.

M-low = the same amount of black soldier fly larvae meal is used as in diet MO-low.

O-low = the same amount of black soldier fly larvae oil is used as in diet MO-low.

L-high = 10% of the daily dry matter feed intake is replaced by live black soldier fly larvae.

MO-high = 10% of the daily dry matter feed intake is replaced by a mix of black soldier fly larvae meal and oil.

M-high = the same amount of black soldier fly larvae meal is used as in diet MO-high.

O-high = the same amount of black soldier fly larvae oil is used as in diet MO-high.

**Table 4:** Proximate composition of frozen black soldier fly larvae (BSF larvae) and BSF larvae meal.

Proximate composition % dry matter	BSF larvae	BSF larvae meal
Moisture content	67.6	6.9
Crude ash	6.2	7.0
True Protein^1^	39.2	46.8
Crude fat	26.9	15.1
Crude fiber	7.7	10.4
N-free substances	15.7	10.9

1 Sum of amino acids analyzed according to Commission Regulation (EC) No 152/2009, 2009 (2009).

**Table 5:** Amino acid composition of frozen black soldier fly larvae (BSF larvae) and BSF larvae meal.

Amino acids % dry matter	BSF larvae	BSF larvae meal
Lysine	2.59	2.99
Methionine	0.68	0.78
Cysteine	0.37	0.43
Aspartic acid	3.73	4.66
Threonine	1.64	2.02
Serine	1.64	2.13
Glutamic acid	5.19	6.06
Proline	2.41	3.18
Glycine	2.35	2.77
Alanine	2.90	3.57
Valine	2.69	3.11
Isoleucine	2.01	2.15
Leucine	3.27	3.72
Tyrosine	2.31	2.70
Phenylalanine	1.70	1.91
Histidine	1.17	1.44
Arginine	1.88	2.52
Tryptophan	0.59	0.73
∑ amino acids	39.20	46.83

**Table 6:** Fatty acid composition expressed in % of total fatty acids of frozen black soldier fly larvae (BSF larvae), BSF larvae meal, and BSF larvae oil.

Fatty acid % of total fatty acids	Molecular formula	BSF larvae	BSF larvae meal	BSF larvae oil
Saturated				
Capric acid	C 10:0	0.95	0.80	1.00
Lauric acid	C 12:0	40.08	34.90	41.10
Myristic acid	C 14:0	9.17	8.30	9.50
Pentadecanoic acid	C 15:0	0.11	<0.1	<0.1
Palmitic acid	C 16:0	15.12	16.30	15.10
Margaric acid	C 17:0	0.19	0.10	0.10
Stearic acid	C 18:0	2.56	3.30	2.50
Arachic acid	C 20:0	0.09	0.10	0.10
Henicosanoic acid	C 21:0	0.05	<0.1	<0.1
Monounsaturated				
Myristoleic acid	C 14:1	0.19	0.20	0.20
Palmitoleinic acid	C 16:1	2.61	3.00	3.00
Cis-vaccenic acid	C 18:1	0.34	0.40	0.40
Oleic acid	C 18:1	9.92	13.30	9.80
Eicosenoic acid	C 20:1	0.11	0.20	1.00
Polyunsaturated				
Linolic acid	C 18:2	16.78	17.20	14.60
alpha-linolenic acid	C 18:3	1.39	1.30	1.20
Eicosapentaenic acid	C 20:5	<0.1	0.20	0.20
∑				
Saturated fatty acids		68.35	63.80	69.40
Monounsaturated fatty acids		13.14	17.10	14.40
Polyunsaturated fatty acids		18.18	18.70	16.00

The symbol “<”. in the result column means that the substance concerned was not quantifiable as it was below the limit of detection.

#### Insect product incorporation into the broiler ration

**Figure 1:**
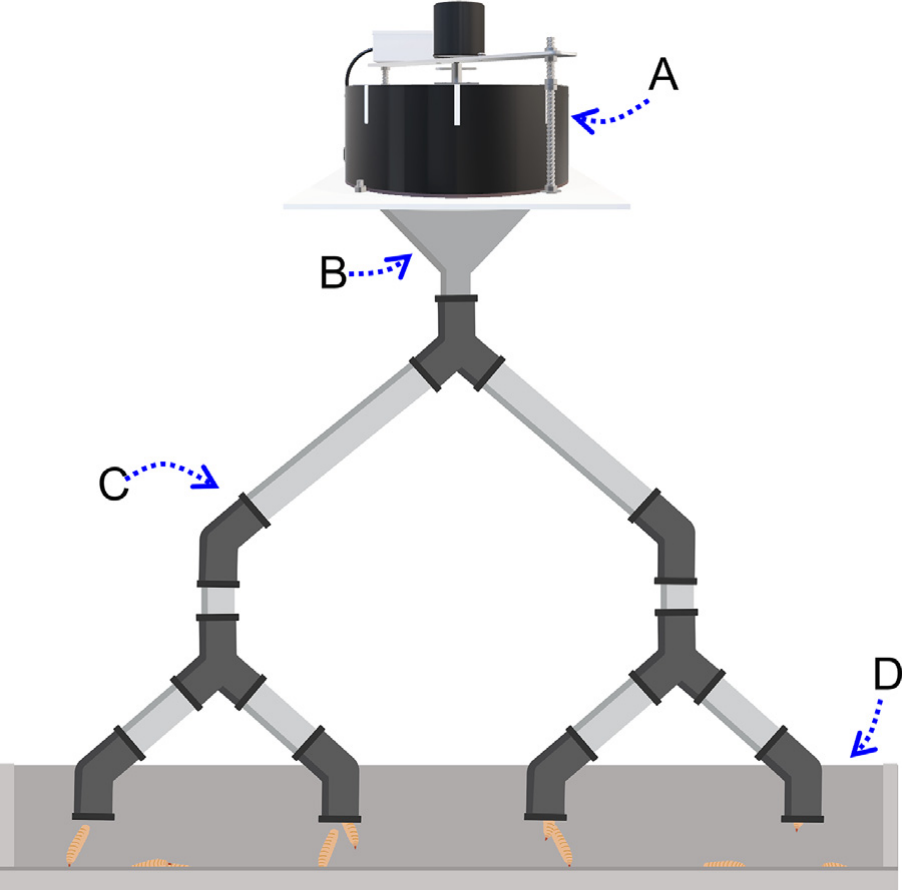
Schematic representation of the larvae dispensing system. Components: A motorized larvae dispenser (Dörper et al., 2023), B Funnel, C Tube system (PVC-U tube, ⌀ 32 mm, width 1.5 mm, AR0032.P08.J01, HT CONNECT GmbH & Co. KG, Pegnitz, Germany) with 4 outlets, D Feed trough (1m long).

The insect meal and oil were incorporated in the diets, while live insects were provided, as such, via a separate feeder next to a complementary diet. A dispenser system (Dörper et al., 2023) was used to gradually provide the live larvae during a period of 8 to 10 h (8 am–6 pm), to facilitate a gradual uptake of larvae by the broilers (Figure 1). The design of the dispenser system was inspired by a feeding equipment (Leushuis and Paul, 2020) used in previous research (Star et al., 2020). The daily portion of live larvae per pen was calculated based on the expected feed intake (P. van Boekholt, pers. com.) and prepared in the morning before filling the dispensers. The larvae feeder was first placed on the floor and over time lifted to the chest height of the growing broilers (Figure 1D).

### Live Larvae Preparation

Live BSF larvae were obtained once a week from Protix BV (Dongen, the Netherlands). After arrival of the live larvae at the research facility, the larvae were stored in crates (60*40*16 cm) each containing approximately 5 kg live larvae and sawdust to keep the larvae dry during the storage. The larvae were kept in a climate-controlled room at 10°C and 70% relative humidity until use. During these storage conditions the metabolic activity and larvae activity were reduced to avoid metabolism and further development of the larvae. Every morning the daily portion of live larvae per pen was prepared: the larvae were separated from the sawdust by using a 4mm sieve. During the preparation process (∼1h) at room temperature the larvae changed from an inactive into an active stage. After sieving, a sample (100 g) was taken from the larvae and stored at -20°C. After the trial, a pooled sample (500 g) of larvae that had been stored at -20°C was analyzed for proximate, amino acid and fatty acid composition (Tables 4, 5, 6).

### Broilers

#### Handling at arrival

The experiment was conducted with 1,728 one-day-old slow-growing male broilers (Hubbard JA757). Broilers were assigned to one of the 9 treatment groups after arrival. Each pen contained 24 animals and each treatment was replicated eight times, leading to a total of 72 pens. Treatments were randomized over pens in 5 different rooms. The experimental design was a randomized complete block design, with room being the block factor and pens were the experimental unit. To achieve a similar body weight per pen at the start of the experiment the chickens were first randomly assigned to one of 72 boxes. After measuring the group weight per box, chickens were exchanged between boxes to achieve that the group weight of 24 chickens per box was equal to the average weight per box of 24 chickens ±3%. Afterwards the chickens were individually weighed and placed into the 72 pens.

#### Housing and management

The experiment was executed at the animal experiment facility Carus of Wageningen University & Research (Wageningen, the Netherlands). The broilers were housed in pens of 2.15 m^2^ (1.95 m x 1.1 m). The pens were distributed over 5 rooms and each equipped with a 1 m perch, seven drinking nipples with drip cups, a round feeder for pellets, a straight 1 m feeder (either empty or for release of live larvae) and wood shavings as bedding material (2 kg/m²). To prevent the broilers from moving between pens over the wired fences, the pens were covered with a metal mesh (mesh size: 5 × 5 cm).

Before arrival of the broilers the rooms were pre-heated to reach a room temperature of 34°C. After the start of the trial the temperature was reduced gradually to 18°C until d 40 and remained at this temperature until the end of the experiment. The relative humidity within the experimental rooms was kept around 50 to 60% during the first week (d 0–6), between 40 to 60% until d 12, and 40 to 70% until the end of the experiment (Table S 15). The light schedule was in alignment with the standard commercial practice for broiler chickens. At arrival of the chickens the artificial light was set to 24 h light, until d 7 the night period was stepwise increased to achieve a light:dark schedule of 18L:6D (Table S 16) and remained at this schedule until the end of the experiment.

The one-day-old chicks were vaccinated at the hatchery against Infectious Bronchitis (spray; Poulvac IB Primer, Zoetis, Capelle aan de IJssel, The Netherlands). At d of arrival, the one-day-old chicks were vaccinated against coccidiosis (spray, Paracox-8, MSD, Boxmeer, The Netherlands). On d 14, all chickens were vaccinated against New Castle Disease (spray, Nobilis ND Clone 30, MSD, Boxmeer, The Netherlands). On d 21, the broilers were additionally vaccinated against Gumboro (spray, Nobilis Gumboro D78, MSD, Boxmeer, The Netherlands).

### Measurements

#### Performance

The performance of the broilers was evaluated weekly per pen. Parameters of interest were the water intake (**WI**), feed intake (FI), body weight (**BW**), body weight gain (**BWG**), and feed conversion ratio (**FCR**). The BW of the broilers was determined individually or on pen level every other week. The individual weighing was performed to determine the uniformity of body weight among broilers within one pen. The last measurement of performance parameters was done on d 49. Mortality was recorded daily.

#### Carcass characteristics

The carcass (defined as the broiler weight after removal of all feathers) and meat (defined as the weight of the breast, drumsticks, and wings) yields of 144 broilers (2 broilers/pen, 16 broilers/treatment) were determined by a commercial slaughterhouse. The following meat yields were determined: empty carcass, breast meat, thighs, drumsticks, and wings. Broilers with a body weight of ±3% of the average broiler body weight per pen were chosen for this measurement. For individual identification of the 144 broilers at the slaughterhouse the animals were tagged prior to transportation with a wing tag. The animals were transported to the slaughterhouse on d 49.

### Statistical Analysis

#### Data preparation

DMFI, cumulative DMFI, cumulative WI, BWG and FCR were calculated as indicated in the equations below. Body weight variation was based on biweekly individual BW data and expressed as standard deviation per pen. The mortality of the broilers was expressed as the percentage of broilers that died (or were removed based on predefined criteria known as humane endpoints to prevent or end an animal's discomfort) per pen of the total number of broilers at the beginning of the experiment per pen.

$$daily\;BWG\;per\;broiler\;per\;week=\frac{body\;weight\;per\;pen\;at\;the\;end\;of\;a\;week}{number\;of\;animals}-\frac{body\;weight\;per\;pen\;at\;the\;start\;of\;a\;week}{number\;of\;animals}$$

$$FC\mathrm{xR}\;broiler\;per\;week\;=\frac{DM\;feed\;input\;\left(pellets+larvae\right)\;per\;pen\;per\;week-DM\;feed\;leftover\;\left(pellets+larvae\right)\;per\;pen\;per\;week}{body\;weight\;per\;pen\;at\;the\;start\;of\;a\;week-body\;weight\;per\;pen\;at\;the\;start\;of\;a\;week\;(including\;weight\;of\;dead\;broilers)\;}$$

$$daily\;DMFI\;per\;broiler\;per\;week\;=\frac{FCR\;per\;broiler\;per\;week*BWG\;per\;broiler\;per\;week}{7\;days}$$

$$Cumulative\;DMFI\;per\;broiler\;per\;day\;=\frac{FCR\;from\;week\;1\;to\;7*\;BWG\;from\;week\;1\;to\;7}{49\;days}$$

$$Cumulative\;WI\;per\;broiler\;per\;day\;=\frac{\;water\;input\;per\;pen\;from\;week\;1\;to\;7\;-water\;remaining\;per\;pen\;from\;week\;1\;to\;7}{number\;of\;animals\;and\;49\;days}$$

$$Carcass\;yield\;per\;broiler\;=\;\frac{carcass\;weight\;}{live\;wieght}*100$$

$$Meat\;yield\;per\;broiler\;=\;\frac{meat\;weight}{carcass\;weight}*100$$

The carcass characteristics were obtained for individual animals, whereas the other response variables were obtained per pen.

#### Models

The analysis of the data was performed by using the open-source software R (R version 4.1.2, R Core Team, 2021). Data files were imported into R with the package “readxl” (Wickham and Bryan, 2019).

Response variables cumulative DMFI and cumulative WI were analyzed with linear mixed effect models (using R library, lme4: Bates et al., 2015) with treatments (control, L-low, MO-low, M-low, O-low, L-high, MO-high, M-high, O-high) having fixed effects and random effects for rooms (5 different rooms).

Response variables DMFI, BWG, BW variation, and FCR were analyzed by linear mixed effect models (using R library, lme4: Bates et al., 2015) with treatments, wk (1 to 7) and the interaction of the two factors having fixed effects. The pen number (1 to 72) was added with random effects to account for repeated measurements and random effects for room. A random effect for weeks nested in rooms was added to the model as well. Dry matter feed intake and BWG were square-root transformed, and FCR and variation in BW were log-transformed to meet the model assumptions.

For the analyses of mortality and carcass characteristics a generalized linear mixed model (GLMM) (using R library glmmTMB: Brooks et al., 2017) was used. The model for mortality employed the beta binomial distribution and the models for carcass characteristics employed the beta distribution for the responses, suitable to analyze proportion data based on counts or continuous numbers (Douma and Weedon, 2019). The models contained the treatment factor with fixed effects and the room factor having random effects. Besides, the model for the carcass characteristics additionally contained factor pen with random effects.

In addition to all models, customized contrasts were defined to test additional linear hypotheses (Bolker; Fox and Weisberg, 2019) for the effects of inclusion level (low and high), diet type (control, live, meal+oil, meal, oil) and the interaction between inclusion level and diet type (excluding the control group). For models with the fixed effects factor week, additional linear hypotheses were tested for the interaction between week and inclusion level, week and diet type, and the three-way interaction between week, inclusion level and diet type. For all models, the model fit was evaluated by visual inspection of the residuals with diagnostic plots (DHARMa: Hartig, 2022). Significance was defined as *P* ≤ 0.05. In the case of significance, multiple comparisons were performed using the multivariate t-distribution method (emmeans: Lenth, 2021, multcomp: Hothorn et al., 2008). P-values of multiple comparisons were adjusted using the multivariate t-distribution method.

The BW measured at wk 3 of 2 pens, one pen of treatment M-low and one pen of treatment L-high, was excluded from analyses. For these pens, an average BW per animal was measured that was 28% and 50% lower than the average BW of animals in other pens of the same treatment. In wk 4 one pen of treatment O-low had a DMFI 40% higher than other pens of the same treatments, due to very low feed leftover at the end of the week. The results of those pens do not correspond with the bidaily visual inspections of the animals and are unlikely to be correctly recorded. The three measurements were therefore excluded from the statistical analyses.

## RESULTS

### Dry Matter Feed Intake

**Figure 2:**
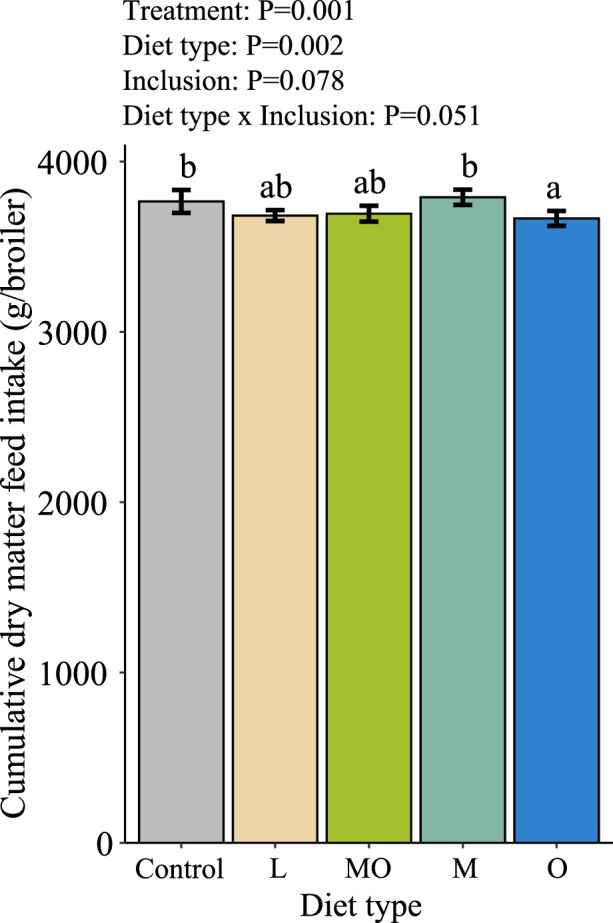
Cumulative dry matter feed intake (g/broiler/d) of slow-growing broilers (Hubbard JA757) during the entire experiment period (0–49 d). Data is presented as mean ± standard error. Different lower case letters indicate significance (*P <* 0.05). The bars indicate the different diet types (Control, L= diets with live larvae, MO= diets with BSF larvae meal and oil, M= diets with BSF larvae meal, and O = diets with BSF larvae oil). The control group was excluded in the contrast of the interaction between diet type and inclusion level.

The DMFI of the broilers increased per week and was influenced by diet type (Table 7). The effect of diet type was dependent on the week. Broilers receiving live larvae had a lower DMFI compared to broilers of the control group in week three and six. Broilers having BSF larvae meal and oil combined in their diet had a lower DMFI compared to broilers of the control group in wk 6 and broilers having BSF larvae oil in their diet had a lower DMFI compared to the control group in wk 6 and 7 and a lower DMFI compared to broilers having BSF larvae meal in their diet at wk 4 to 6. Broilers having BSF larvae meal in their diet had a comparable DMFI compared to broilers of the control group over the entire experimental period of 7 wk. Inclusion level of the BSF larvae products did not influence the DMFI. There was also no interaction between inclusion level and week, nor a 3-way interaction between diet type, inclusion level and week for the DMFI. Moreover, diet type had a significant effect on the cumulative DMFI (Figure 2). Broilers fed with diets containing BSF larvae oil as the only BSF larvae product in the diet had a lower cumulative DMFI compared to broilers of the control group and broilers fed with BSF larvae meal in their diet.

**Table 7:** Daily dry matter feed intake (g/broiler/d, DM of pellets and live larvae) per wk of slow-growing broilers (Hubbard JA757) during 7 wk of growth.

Week (broiler age in d)	Control	L	MO	M	O	mean
1 (1–7)	13.5 ± 0.2	13.0 ± 0.2	13.4 ± 0.2	13.4 ± 0.2	13.3 ± 0.2	13.3 ± 0.1^A^
2 (8–14)	32.3 ± 0.9	31.7 ± 0.6	32.3 ± 0.6	32.3 ± 0.6	31.4 ± 0.5	32.0 ± 0.3^B^
3 (15–21)	53.5 ± 1.5^b^	50.7 ± 0.9^a^	52.7 ± 1.0^ab^	54.4 ± 0.8^b^	51.4 ± 1.0^ab^	52.4 ± 0.5^C^
4 (22–28)	77.6 ± 1.9^ab^	75.6 ± 1.1^a^	76.3 ± 1.4^a^	80.0 ± 1.3^b^	75.5 ± 1.2^a^	77.0 ± 0.6^D^
5 (29–35)	98.9 ± 1.5^ab^	96.9 ± 1.2^ab^	97.6 ± 1.3^ab^	100.8 ± 1.4^b^	96.4 ± 1.4^a^	98.0 ± 0.6^E^
6 (36–42)	124.4 ± 2.5^c^	120.5 ± 1.2^ab^	119.6 ± 1.6^a^	124.2 ± 1.5^bc^	119.2 ± 1.6^a^	121.3 ± 0.8^F^
7 (43–49)	138.4 ± 1.6^b^	138.0 ± 0.8^ab^	136.8 ± 1.9^ab^	138.0 ± 1.4^ab^	134.9 ± 1.7^a^	137.1 ± 0.7^G^
						
	***P*-value^5^**	***Degrees of freedom***				
**Treatment**^1^	**<0.001**	8				
**Diet type**^2^	**0.001**	4				
Inclusion^3^	0.105	1				
Diet type*Inclusion^4^	**0.039**	3				
**Week**	**<0.001**	6				
Treatment*Week	**0.011**	48				
Diet type*Week	**0.003**	24				
Inclusion*Week	0.422	6				
Diet type*Inclusion*Week	0.265	18				

Data is presented as mean ± standard error.

Different lower-case letters in a row or upper-case letters in a column indicate significance (*P <* 0.05).

1 Treatments (Control = Conventional corn-wheat based broiler diet, L-low = 5% of the daily dry matter feed intake is replaced by live black soldier fly larvae, MO-low = 5% of the daily dry matter feed intake is replaced by a mix of black soldier fly larvae meal & oil, M-low = the same amount of black soldier fly larvae meal is used as in diet MO-low, O-low = the same amount of black soldier fly larvae oil is used as in diet MO-low, L-high = 10% of the daily dry matter feed intake is replaced by live black soldier fly larvae, MO-high = 10% of the daily dry matter feed intake is replaced by a mix of black soldier fly larvae meal & oil, M-high = the same amount of black soldier fly larvae meal is used as in diet MO-high, O-high = the same amount of black soldier fly larvae oil is used as in diet MO-high). Every treatment had eight replicates.

2 Diet types (Control, L= diets with live larvae, MO= diets with BSF larvae meal and oil, M= diets with BSF larvae meal, and O = diets with BSF larvae oil).

3 Inclusion (low= diets with low inclusion of BSF larvae products, and high =diets with high inclusion of BSF larvae products).

4 The control group was excluded in the contrast of the interaction between diet type and inclusion level.

### Water Intake

**Figure 3:**
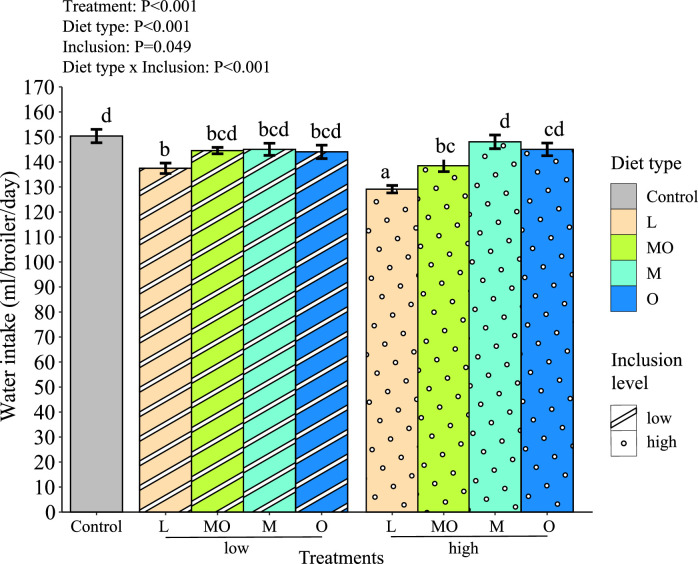
Cumulative water intake (mL/broiler/d) from the nipple drinkers by slow-growing broilers during the entire experiment period (0–49 d). Data is presented as mean ± standard error. Different lower-case letters indicate significance (*P <* 0.05) between different treatments (Control = Conventional corn-wheat based broiler diet, L-low = 5% of the daily dry matter feed intake is replaced by live black soldier fly larvae, MO-low = 5% of the daily dry matter feed intake is replaced by a mix of black soldier fly larvae meal and oil, M-low = the same amount of black soldier fly larvae meal is used as in diet MO-low, O-low = the same amount of black soldier fly larvae oil is used as in diet MO-low, L-high = 10% of the daily dry matter feed intake is replaced by live black soldier fly larvae, MO-high = 10% of the daily dry matter feed intake is replaced by a mix of black soldier fly larvae meal and oil, M-high = the same amount of black soldier fly larvae meal is used as in diet MO-high, O-high = the same amount of black soldier fly larvae oil is used as in diet MO-high). Every treatment had 8 replicates. The control group was excluded in the contrast of the interaction between diet type and inclusion level.

The cumulative WI of broilers was influenced by several factors (Figure 3) such as the interaction between diet type and inclusion level. Broilers fed with live larvae, or a high inclusion level of larvae meal and oil combined in their diet consumed less water from the nipple drinkers than broilers of the control group. At the high level of live larvae provision, water consumption by the broilers from the nipple drinkers was lower compared to the low level of live larvae provision. BSF larvae meal and oil provided separately in the broiler diet did not alter broiler WI, regardless of the inclusion level of the BSF larvae products in the diets.

### Body Weight Gain and Body Weight Variation

The BWG of the broilers increased over the weeks and remained stable over the last 2 wk of the experiment. Diet type significantly influenced the BWG of the broilers (Table 8). Broilers receiving live larvae and larvae meal had a higher BWG compared to broilers fed with BSF larvae oil in their diets. However, none of the diet types led to altered BWG compared to the control group. No effects of inclusion level and the interaction between diet types and inclusion levels on BWG were detected.

**Table 8:** Daily body weight gain (g/broiler/wk) of slow-growing broilers (Hubbard JA757) during 7 wk of growth.

Week (broiler age in d)	Control	L	MO	M	O	mean
1 (1–7)	14.46 ± 0.29	14.30 ± 0.26	14.44 ± 0.23	14.68 ± 0.21	14.11 ± 0.21	14.39 ± 0.11^A^
2 (8–14)	28.55 ± 0.90	28.40 ± 0.56	28.26 ± 0.62	28.33 ± 0.58	27.64 ± 0.48	28.20 ± 0.26^B^
3 (15–21)	40.64 ± 1.41	40.21 ± 0.84	40.96 ± 0.92	41.98 ± 0.58	39.80 ± 0.90	40.72 ± 0.40^C^
4 (22–28)	55.95 ± 1.17	56.08 ± 0.71	56.21 ± 0.90	58.93 ± 1.12	55.59 ± 0.91	56.59 ± 0.44^D^
5 (29–35)	65.79 ± 0.76	66.64 ± 0.81	65.79 ± 0.88	67.54 ± 0.97	65.09 ± 1.09	66.21 ± 0.43^E^
6 (36–42)	78.46 ± 1.30	78.50 ± 1.04	76.42 ± 1.05	78.92 ± 0.87	75.92 ± 1.03	77.55 ± 0.48^F^
7 (43–49)	79.83 ± 0.79	81.11 ± 0.66	80.76 ± 1.19	80.09 ± 1.07	79.17 ± 0.60	80.23 ± 0.41^F^
**mean**	51.96 ± 3.15^ab^	52.25 ± 2.27^b^	51.83 ± 2.21^ab^	52.97 ± 2.26^b^	51.05 ± 2.19^a^	
	***P*-value**	***Degrees of freedom***				
**Treatment**^1^	**0.008**	8				
**Diet type**^2^	**0.007**	4				
Inclusion^3^	0.933	1				
Diet type*Inclusion^4^	0.063	3				
**Week**	**<0.001**	6				
Treatment*Week	0.426	48				
Diet type*Week	0.442	24				
Inclusion*Week	0.698	6				
Diet type*Inclusion*Week	0.258	18				

Data is presented as mean ± standard error.

P-values < 0.05 are highlighted in bold.

Different lower-case letters in a row or upper-case letter in a column indicate significant differences (*P <* 0.05).

1 Treatments (Control = Conventional corn-wheat based broiler diet, L-low = 5% of the daily dry matter feed intake is replaced by live black soldier fly larvae, MO-low = 5% of the daily dry matter feed intake is replaced by a mix of black soldier fly larvae meal & oil, M-low = the same amount of black soldier fly larvae meal is used as in diet MO-low, O-low = the same amount of black soldier fly larvae oil is used as in diet MO-low, L-high = 10% of the daily dry matter feed intake is replaced by live black soldier fly larvae, MO-high = 10% of the daily dry matter feed intake is replaced by a mix of black soldier fly larvae meal & oil, M-high = the same amount of black soldier fly larvae meal is used as in diet MO-high, O-high = the same amount of black soldier fly larvae oil is used as in diet MO-high). Every treatment had eight replicates.

2 Diet types (Control, L= diets with live larvae, MO= diets with BSF larvae meal and oil, M= diets with BSF larvae meal, and O = diets with BSF larvae oil).

3 Inclusion (low = diets with low inclusion of BSF larvae products, and high = diets with high inclusion of BSF larvae products).

4 The control group was excluded in the contrast of the interaction between diet type and inclusion level.

The variation in BW between broilers housed within one pen changed over time significantly. With increasing age of the broilers, the variation in BW increased. The variation of the BW was not significantly influenced by treatment, diet type, and inclusion level, nor any factor interactions (Figure S 1).

### Feed Conversion Ratio

**Figure 4:**
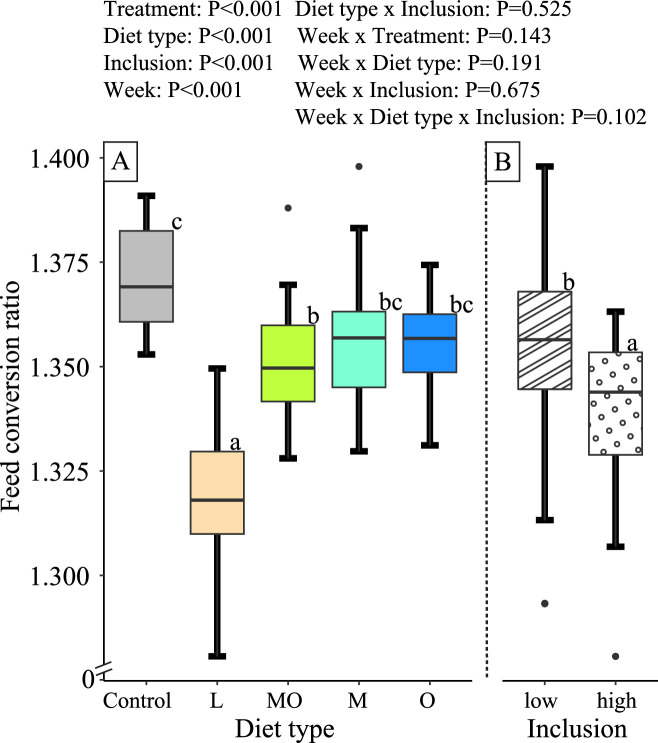
Box plots of the average weekly feed conversion ratio per broiler. Different letters between groups indicate significance (*P* < 0.05). The lower whisker of the boxplots represents the smallest observation ≥25% quantile – 1.5 × interquartile range (IQR). The upper whisker represents the largest observation ≤75% quantile + 1.5 × IQR. Points outside the whiskers represent outliers. Different lower case letters in a figure indicate significance (*P* < 0.05). Boxplots in Figure A indicate the different diet types (Control, L= diets with live larvae, MO= diets with BSF larvae meal and oil, M= diets with BSF larvae meal, and O = diets with BSF larvae oil) and boxplots in Figure B indicate different inclusions (low= diets with low inclusion of BSF larvae products, and high =diets with high inclusion of BSF larvae products). The control group was excluded in the contrast of the interaction between diet type and inclusion level.

The FCR of broilers increased every week and was influenced by diet type. Broilers that received live larvae had the lowest FCR, followed by broilers fed a combination of BSF larvae meal and oil in their diet (Figure 4A). Broilers receiving BSF larvae meal or BSF larvae oil separately in their diet showed intermediate values between the control and diets with BSF larvae oil and meal combined, while broilers of the control group had the highest FCR. The effect of diet type on FCR was independent of the inclusion level of the BSF larvae products. Moreover, higher inclusion levels of the BSF larvae products in the diets led to lower FCR than lower inclusion levels (Figure 4B). The effect of diet type and inclusion level on the broiler FCR was consistent over the experimental time of 7 wk, since no significant interaction of the factors with week was detected.

### Mortality

The mortality was not significantly influenced by treatment (*P =* 0.749), diet type (*P =* 0.850), inclusion level (*P =* 0.189) or the interaction between diet type and inclusion level (*P =* 0.474). On average 0.89 ± 0.12se broiler per pen died or was removed (based on humane endpoints) during the total experimental period of 7 wk.

### Carcass Characteristics

Before slaughter, the live weight of all broilers within a pen and the individual live weight of selected broilers chosen for slaughter were determined. The live weight of the broilers after 7 wk of growth was dependent on the diet type fed (Table 9). Broilers receiving BSF larvae oil were on average 71 and 87g lighter compared to broilers fed with live BSF larvae and BSF larvae meal, respectively. Broilers fed with live BSF larvae and BSF larvae meal had, however, no different live weight compared to broilers of the control group. The analysis of the live weight of the selected broilers led to the same results. For the evaluation of the carcass characteristics the information of 142 instead of 144 animals was available, because two animals lost their wing tag during transport to the slaughterhouse. The carcass, thighs, drumstick, and wing yield were not significantly different between broilers that received different treatments, diet types, nor different inclusion levels of larvae products after 7 wk of growth (Table 9). However, breast yield was significantly influenced by diet type. Broilers that received BSF larvae oil in their diet had a lower breast yield compared to broilers that received live larvae (*P =* 0.013). None of the diet types led to different breast yield compared to the control group. Besides, inclusion level influenced the breast yield. Broilers with low inclusion of BSF larvae products in the diets had lower breast yields compared to broilers with high inclusion of BSF larvae products in the diet. The interaction between diet type and inclusion level was not significant for any of the meat yield parameters.

**Table 9:** Effect of the dietary treatment on live weight, carcass and meat yields of 7-wk-old slow-growing broilers (Hubbard JA757).

	LWa^1^ g/ broiler	LWs^2^ g/ broiler	Carcass yield as % of LWs	Breast yield as % of CW^3^	Thigh yield as % of CW	Drumstick yield as % of CW	Wing yield as % of CW
Diet type							
Control	2587 ± 36^ab^	2613 ± 36^ab^	68.68 ± 0.46	25.58 ± 0.29^ab^	18.33 ± 0.20	14.39 ± 0.16	11.43 ± 0.11
L	2600 ± 18^b^	2630 ± 14^b^	68.99 ± 0.24	26.32 ± 0.31^b^	18.87 ± 0.17	14.41 ± 0.11	11.63 ± 0.09
MO	2582 ± 28^ab^	2608 ± 25^ab^	68.75 ± 0.29	25.69 ± 0.21^ab^	18.59 ± 0.15	14.39 ± 0.10	11.62 ± 0.06
M	2630 ± 27^b^	2646 ± 20^b^	68.92 ± 0.26	25.59 ± 0.30^ab^	18.41 ± 0.13	14.43 ± 0.11	11.64 ± 0.10
O	2543 ± 26^a^	2559 ± 21^a^	68.53 ± 0.25	25.02 ± 0.32^a^	18.54 ± 0.17	14.59 ± 0.11	11.46 ± 0.11
Inclusion							
Low	2589 ± 16	2618 ± 13	68.82 ± 0.19	25.37 ± 0.23^a^	18.68 ± 0.11	14.48 ± 0.08	11.64 ± 0.06
High	2588 ± 21	2603 ± 16	68.78 ± 0.17	25.94 ± 0.18^b^	18.53 ± 0.11	14.44 ± 0.07	11.53 ± 0.07
		*P*-values					
Treatment^4^	0.007	0.011	0.889	0.009	0.364	0.440	0.294
Diet type^5^	0.007	0.004	0.804	0.025	0.161	0.705	0.267
Inclusion^6^	0.941	0.297	0.901	0.028	0.391	0.770	0.233
Diet type*Inclusion^7^	0.051	0.293	0.592	0.159	0.711	0.135	0.372

Data is presented as mean ± standard error.

Different lower-case letters in one column indicate significance (*P* < 0.05) between different treatment groups.

1 Lwa = live weight of all broilers (calculations are based on the group body weight of 20–24 broilers per pen).

2 LWs = live weight of broilers slaughtered to measure carcass characteristics (calculations are based on the individual body weight of two broilers per pen).

3 CW = carcass weight.

4 Treatments (Control = Conventional corn-wheat based broiler diet, L-low = 5% of the daily dry matter feed intake is replaced by live black soldier fly larvae, MO-low = 5% of the daily dry matter feed intake is replaced by a mix of black soldier fly larvae meal & oil, M-low = the same amount of black soldier fly larvae meal is used as in diet MO-low, O-low = the same amount of black soldier fly larvae oil is used as in diet MO-low, L-high = 10% of the daily dry matter feed intake is replaced by live black soldier fly larvae, MO-high = 10% of the daily dry matter feed intake is replaced by a mix of black soldier fly larvae meal & oil, M-high = the same amount of black soldier fly larvae meal is used as in diet MO-high, O-high = the same amount of black soldier fly larvae oil is used as in diet MO-high).

5 Diet types (Control, L= diets with live larvae, MO= diets with BSF larvae meal and oil, M= diets with BSF larvae meal, and O = diets with BSF larvae oil).

6 Inclusion (low= diets with low inclusion of BSF larvae products, and high =diets with high inclusion of BSF larvae products).

7 The control group was excluded in the contrast of the interaction between diet type and inclusion level.

## DISCUSSION

This study is the first comparing the effects of unprocessed (live) and processed (meal + oil, meal, and oil) BSF larvae in broiler diets at two inclusion levels on performance and carcass characteristics within one study. When comparing feed intake and FCR results with other studies, it is important to note that the results are presented on a dry matter basis. This approach may be different from most literature but was necessary due to the inclusion of live larvae, which contain 67.6% moisture.

Performance parameters were affected by the BSF larvae product used in the diet. The inclusion of BSF larvae oil in the broiler diet led to a 3.6% lower BWG and 3.3% lower cumulative DMFI compared to broilers fed with BSF larvae meal in their diet. The combination of both products in one diet led to intermediate results for the BWG and cumulative DMFI. Yet, even though the FCR was the same for broilers of the control and broilers fed BSF larvae meal or BSF larvae oil, the inclusion of a combination of both products in one diet resulted in a lower FCR compared to the control. It appears that the broilers’ performance is best when both products are combined in one diet. Similar results were found in previous research, where the inclusion of BSF larvae meal (Dabbou et al., 2018; Mutisya et al., 2021; Machado et al., 2023) or BSF larvae oil (Schiavone et al., 2018; Dabbou et al., 2021; Schäfer et al., 2023) in broiler diets did not significantly affect their FCR, while the combination of both products in the diet was found to improve broiler FCR (Facey et al., 2023). Broilers fed diets in which 8 to 10 % of the DMFI was replaced by BSF larvae meal and oil showed a reduced FCR compared to the control group during the starter and grower phase. In contrast to the current study, Facey et al. (2023) focused on the replacement of a certain percentage of soybean meal in the diet. Since soybean meal inclusion varied across diets of different developmental phases for the broilers, also the inclusion of BSF larvae meal in the diet varied per feeding phase. Moreover, the ratio between BSF larvae meal and oil in the diets changed per feeding phase (Facey et al., 2023). The effect of the dietary treatment might have been only temporarily or feeding-phase related, since the percent inclusion and ratio of the insect products fluctuated over time. To put the results of our current study into perspective, diet programs in which 10% of the DMFI was replaced by live BSF larvae or meal and oil combined, translates to replacement of 26-35% of the soybean meal in the diets depending on the feeding phase. This information should be taken with caution as ingredient levels vary considerably per feed formulation. Inclusion levels expressed as a percentage of soybean meal or soybean oil replacement in the diet are therefore not a reliable unit to directly compare study outputs across studies nor to evaluate the economic or environmental context.

The positive effect of feeding live BSF larvae or BSF meal and oil combined on the FCR might be based on the presence of different bioactive compounds in the larvae products within the broiler diets. It has been suggested that lauric acid, chitin, and antimicrobial peptides (Gasco et al., 2018; Dörper et al., 2021) in BSF products can shape the intestinal microbiome by suppressing pathogenic bacteria and supporting beneficial bacteria (Skrivanova et al., 2006; Timbermont et al., 2010; Choi et al., 2012; Wang et al., 2017; Zheng et al., 2018; Wu et al., 2021). Previous research has highlighted the relationship between the FCR and the intestinal microbial community (Rinttilä and Apajalahti, 2013; Shah et al., 2019). Thereby, the absence of potential pathogens might allow the use of nutrients for growth rather than for pathogen defense or inflammatory responses. Moreover, other bacteria might contribute to host performance by providing nutrients. For instance, bacteria of the genera *Bacteriodes, Megamonas, Acidaminococcus, Prevotella,* and *Paraprevotella* are commonly part of the microbial community in the cecum of broilers and are known to ferment dietary fiber, such as chitin, into short chain fatty acids (SCFA, Shah et al., 2019). Those SCFA can then be used by the host as an energy source, for instance for colonic epithelial cells (Roediger, 1982; Rinttilä and Apajalahti, 2013; Shah et al., 2019). It can be assumed that the more bioactive compounds are present in the diet, the greater is their impact on these processes. The results support this, as inclusion level of the BSF larvae products impacted the FCR. The more BSF larvae products were included, the lower was the FCR. Moreover, additional processing of the larvae may affect the presence and functionality of bioactive compounds. Various processing techniques have been patented and applied in industry to obtain meal and oil products from the intact BSF larvae (Ravi et al., 2020; Smets, 2021). The effect of processing steps such as heat treatment or enzymatic hydrolysis on the bioactive compounds have to the best of our knowledge not been investigated yet. The application of these processing steps to the intact larvae leads to the separation of nutrients and therefore bioactive compounds. For instance, BSF larvae oil contains more lauric acid than BSF larvae meal. The results of the current study point out that a combination of the BSF larvae products and therefore bioactive compounds benefit broiler FCR. This is further supported by the FCR of the broilers fed with live larvae. The live larvae represent the least processed larvae product tested, generating the best FCR. It should be noted that the analysis of the pooled larvae samples collected during the broiler trial showed a true protein (sum of amino acids analyzed according to Commission Regulation (EC) No 152/2009, 2009, 2009) content of 39.2% DM and a crude fat content of 26.9 % DM. It was expected that the fat content of the larvae would be similar to the protein content (Barragán-Fonseca et al., 2017). In light of this finding, it is even more remarkable that live BSF larvae fed broilers showed the lowest FCR, as fat is an important source for energy. The reason for the lower fat content of the larvae was not further investigated, but could be caused due to transport or storage of the larvae. After harvest, the larvae were transported to the research facility and kept in cold storage in sawdust without feed until use for a maximum of 1 wk. To our knowledge there is no information available about the effect of short-term cold storage on the nutritional quality of live insects used as feed. However, storage of parasitic wasp larvae in aphid mummies at 2 and 4°C for 3 wk led to a loss of fresh matter, moisture, dry matter, and fat. The larvae were expected to enter dormancy during the storage conditions. Nevertheless, the parasitoid larvae depleted fat reserves even though metabolism was slowed down (Colinet et al., 2006). To which extent this applies to BSF larvae stored for 7 d at low temperatures until further use as feed should be investigated.

Unlike the effects on FCR, neither treatments, diet types, nor inclusion levels led to significant differences in LW at the end of the trial, carcass or meat yields compared to broilers of the control group. This is in line with previous research using BSF larvae meal (Schiavone et al., 2019; Machado et al., 2023), and BSF larvae oil (Schiavone et al., 2018) in broiler diets. The results reassure that all BSF larvae products in broiler diets result in favorable commercial output.

Regarding the variation in BW, it was expected that in pens receiving live larvae, hierarchical structures within the flock might cause unequal uptake of live larvae, leading to unbalanced distribution of nutrients and thereby higher variation in body weight in those pens compared to pens with broilers only receiving pelleted feed. This assumption was based on the fact that hierarchy in broiler flocks plays an important role in feed intake. When feed is limited or presented in small portions, dominant animals might limit the access to feed to subordinate individuals (Nielsen et al., 2023). In addition, poultry is naturally attracted to live larvae (Veldkamp and Van Niekerk, 2019; Star et al., 2020). As an example, turkeys provided with live larvae once per day consumed the daily live larvae portion within 2 min, highlighting their eagerness to eat live larvae (Veldkamp and Van Niekerk, 2019). Against the expectation, flock uniformity measured as variation in body weight was neither influenced by treatment nor by diet type. This is indicative of a balanced uptake of live larvae across broilers within a pen. Because the larvae were provided by a device that was developed to avoid that the larvae were provided all at once, but over several hours (Dörper et al., 2023), this likely has enabled all broilers to take up larvae so there was no difference in BW uniformity. Live larvae have a large volume because they contain considerable amounts of moisture (Table 4). Possibly broilers that consumed live larvae did not attempt to consume them in the subsequent moment of release due to satiation, allowing broilers that did not obtain larvae at the previous opportunity to consume them at the next.

The results of our study show that the cumulative WI was influenced by the inclusion of BSF larvae products in the broiler diets. Provision of live larvae led on average to 11.4% lower water consumption from the drinkers compared to the control group. Furthermore, at the high provision level of live larvae less water was consumed by the broilers. This is in line with studies focusing on broiler behavior. The drinking pattern of broilers was influenced by the inclusion of live larvae, which resulted in broilers spending less time drinking (Ipema et al., 2020a,^2022^). Live larvae in the current study contained 67.6% moisture, comparable to data of previous studies reporting 67.7- 70.0% (Veldkamp and Van Niekerk, 2019; Star et al., 2020). Since a considerable quantity of water is taken up via the larvae, the broilers adjusted their drinking habits by reducing their water consumption at the drinkers. The water footprint (m^3^ water/ ton meat) of poultry meat production is lower in comparison to other meat production systems such as pork and beef (Mekonnen and Hoekstra, 2012; Gerbens-Leenes et al., 2013). Our data indicate that feeding live larvae to broilers has the potential to further reduce the water footprint during production. Moreover, drinkers in the poultry barn are a source of water spillage and wet litter (Ekstrand et al., 1997; Lynn and Elson, 1990; Nicholson et al., 2004), which can in turn lead to ammonia emission (Nicholson et al., 2004) and prevalence of footpad dermatitis (Ekstrand et al., 1997; Mayne, 2005) and hock burns (Tucker and Walker, 1992; Mayne, 2005). Feeding live larvae could reduce the environmental effect and improve broiler welfare during production. It is important to consider that in practice water intake is used as a tool to monitor broiler performance and health (DEFRA, 2002; Manning et al., 2007). The water intake of broilers fed conventional diets corresponds to ∼1.75 times the feed intake (P. van Boekholt, pers. com.). This is lower when including live larvae in broiler diets.

In conclusion, the current study highlights that BSF larvae products are valuable feed ingredients ensuring good performance and carcass characteristics. Comparable or better results were achieved for those parameters with the BSF larvae products in the diets compared to the control. Based on the FCR the least processed larvae product, that is, live larvae, and the highest inclusion level led to the most favorable results. BW uniformity indicates that the feeding method used secured equal uptake of live larvae across the broilers fed with live larvae. Moreover, while processed larvae products function as a source for nutrients, live larvae also function as a source for moisture leading to a lower water intake from the drinkers. Future research should address the effect of live larvae storage on their nutritional composition. Standardization of research methods is recommended to make research output of studies using insect products for poultry nutrition more comparable.

## Appendix Supplementary materials

Image, application 1

## Footnotes

Supplementary material associated with this article can be found in the online version at doi:10.1016/j.psj.2024.103481.
