# Supplementary material for: Effects of different black soldier fly larvae products on slow-growing broiler performance and carcass characteristics
Source: Poult Sci. 2024 Jan 19;103(4):103481. doi: 10.1016/j.psj.2024.103481 (PMC10869907; doi:10.1016/j.psj.2024.103481)
Supplement: Supplementary file 1 [file mmc1.docx]

# Supplementary information

**Table S 1**: Nutrient composition of pelleted starter feed per experimental treatment

| **Calculated nutrients**  **(g/kg as-fed, unless declared otherwise)** | **Control** | **L-**  **low** | **MO-**  **low** | | **M-**  **low** | **O-**  **low** | **L-**  **high** | **MO-**  **high** | **M-**  **high** | **O-**  **high** | |
| --- | --- | --- | --- | --- | --- | --- | --- | --- | --- | --- | --- |
| Dry matter | 886.27 | 883.36 | 886.81 | | 886.75 | 886.40 | 880.33 | 887.54 | 887.33 | 886.50 | |
| Crude protein | 191.87 | 186.97 | 195.52 | | 196.00 | 191.79 | 183.89 | 201.10 | 201.22 | 191.73 | |
| Crude fat | 75.72 | 52.89 | 71.55 | | 70.63 | 77.17 | 28.69 | 68.62 | 66.13 | 78.41 | |
| Crude fiber | 26.29 | 26.46 | 28.48 | | 28.54 | 26.26 | 26.79 | 30.79 | 30.85 | 26.23 | |
| Ash | 63.50 | 64.30 | 63.19 | | 63.27 | 63.49 | 65.29 | 63.19 | 63.20 | 63.48 | |
| Cl | 2.00 | 1.99 | 2.00 | | 2.00 | 2.00 | 2.02 | 2.00 | 2.00 | 2.00 | |
| K | 8.16 | 7.80 | 7.82 | | 7.85 | 8.16 | 7.56 | 7.63 | 7.63 | 8.16 | |
| Mg | 1.49 | 1.46 | 1.50 | | 1.51 | 1.49 | 1.45 | 1.54 | 1.54 | 1.48 | |
| Ca | 9.00 | 9.25 | 9.00 | | 9.00 | 9.00 | 9.44 | 9.00 | 9.00 | 9.00 | |
| Na | 1.60 | 1.63 | 1.60 | | 1.60 | 1.60 | 1.65 | 1.60 | 1.60 | 1.60 | |
| P | 6.56 | 6.82 | 6.85 | | 6.85 | 6.56 | 7.13 | 7.15 | 7.15 | 6.56 | |
| Retainable phosphorus (rP) | 4.00 | 4.22 | 4.00 | | 4.00 | 4.00 | 4.48 | 4.00 | 4.00 | 4.00 | |
| Ca : rP | 2.25 | 2.19 | 2.25 | | 2.25 | 2.25 | 2.11 | 2.25 | 2.25 | 2.25 | |
| Metabolizable energy (MJ) | 12.33 | 11.89 | 12.39 | | 12.34 | 12.38 | 11.40 | 12.43 | 12.35 | 12.42 | |
| Digestible lysine | 12.30 | 12.13 | 12.30 | | 12.30 | 12.30 | 11.96 | 12.30 | 12.30 | 12.30 | |
| Digestible methionine | 6.54 | 6.83 | 6.68 | | 6.67 | 6.54 | 7.19 | 6.78 | 6.78 | 6.54 | |
| Digestible cysteine | 2.56 | 2.49 | 2.42 | | 2.43 | 2.56 | 2.44 | 2.32 | 2.32 | 2.56 | |
| Digestible methionine + cysteine | 9.10 | 9.32 | 9.10 | | 9.10 | 9.10 | 9.63 | 9.10 | 9.10 | 9.10 | |
| Digestible threonine | 7.80 | 7.67 | 7.80 | | 7.80 | 7.80 | 7.48 | 7.80 | 7.80 | 7.80 | |
| Digestible tryptophane | 1.90 | 1.79 | 1.90 | | 1.90 | 1.90 | 1.72 | 1.90 | 1.90 | 1.90 | |
| Digestible arginine | 12.70 | 12.69 | 12.70 | | 12.70 | 12.70 | 12.76 | 12.70 | 12.70 | 12.70 | |
| Digestible valine | 9.20 | 8.73 | 9.20 | | 9.20 | 9.20 | 8.22 | 9.20 | 9.20 | 9.20 | |
| Linoleic acid C 18:2 | 40.54 | 28.23 | 30.00 | | 36.00 | 34.82 | 15.17 | 20.11 | 31.77 | 28.99 | |
| Control = Conventional corn-wheat based broiler diet  L-low = 5% of the daily dry matter feed intake is replaced by live black soldier fly larvae  MO-low = 5% of the daily dry matter feed intake is replaced by a mix of black soldier fly larvae meal & oil  M-low = the same amount of black soldier fly larvae meal is used as in diet MO-low  O-low = the same amount of black soldier fly larvae oil is used as in diet MO-low | | | | L-high = 10% of the daily dry matter feed intake is replaced by live black soldier fly larvae  MO-high = 10% of the daily dry matter feed intake is replaced by a mix of black soldier fly larvae meal & oil  M-high = the same amount of black soldier fly larvae meal is used as in diet MO-high  O-high = the same amount of black soldier fly larvae oil is used as in diet MO-high | | | | | | |  |

**Table S 2**: Nutrient composition of pelleted grower feed per experimental treatment

| **Calculated nutrients**  **(g/kg as-fed, unless declared otherwise)** | **Control** | **L-**  **low** | **MO-**  **low** | | **M-**  **low** | **O-**  **low** | **L-**  **high** | **MO-**  **high** | **M-**  **high** | **O-**  **high** | |
| --- | --- | --- | --- | --- | --- | --- | --- | --- | --- | --- | --- |
| Dry matter | 886.67 | 883.86 | 887.29 | | 887.19 | 886.77 | 880.74 | 887.91 | 887.70 | 886.88 | |
| Crude protein | 181.95 | 177.91 | 186.65 | | 186.67 | 181.94 | 173.11 | 191.34 | 191.39 | 181.93 | |
| Crude fat | 98.75 | 75.73 | 93.34 | | 93.10 | 98.99 | 50.84 | 88.46 | 87.44 | 99.24 | |
| Crude fiber | 28.09 | 28.48 | 30.39 | | 30.39 | 28.09 | 28.88 | 32.68 | 32.70 | 28.08 | |
| Ash | 60.09 | 60.65 | 59.96 | | 59.95 | 60.10 | 61.44 | 59.82 | 59.81 | 60.10 | |
| Cl | 2.00 | 2.00 | 2.00 | | 2.00 | 2.00 | 2.01 | 2.00 | 2.00 | 2.00 | |
| K | 7.59 | 7.27 | 7.32 | | 7.32 | 7.59 | 6.92 | 7.05 | 7.05 | 7.58 | |
| Mg | 1.42 | 1.40 | 1.45 | | 1.45 | 1.42 | 1.38 | 1.47 | 1.48 | 1.42 | |
| Ca | 8.50 | 8.66 | 8.50 | | 8.50 | 8.50 | 8.86 | 8.50 | 8.50 | 8.50 | |
| Na | 1.60 | 1.61 | 1.60 | | 1.60 | 1.60 | 1.65 | 1.60 | 1.60 | 1.60 | |
| P | 6.27 | 6.50 | 6.57 | | 6.57 | 6.27 | 6.78 | 6.86 | 6.86 | 6.27 | |
| Retainable phosphorus (rP) | 3.78 | 3.97 | 3.78 | | 3.78 | 3.78 | 4.21 | 3.78 | 3.78 | 3.78 | |
| Ca : rP | 2.25 | 2.18 | 2.25 | | 2.25 | 2.25 | 2.11 | 2.25 | 2.25 | 2.25 | |
| Metabolizable energy (MJ) | 12.60 | 12.21 | 12.68 | | 12.62 | 12.66 | 11.76 | 12.75 | 12.64 | 12.72 | |
| Digestible lysine | 11.00 | 10.81 | 11.00 | | 11.00 | 11.00 | 10.55 | 11.00 | 11.00 | 11.00 | |
| Digestible methionine | 5.94 | 6.15 | 6.05 | | 6.06 | 5.93 | 6.38 | 6.15 | 6.17 | 5.92 | |
| Digestible cysteine | 2.49 | 2.44 | 2.37 | | 2.37 | 2.49 | 2.37 | 2.25 | 2.25 | 2.49 | |
| Digestible methionine + cysteine | 8.40 | 8.57 | 8.40 | | 8.40 | 8.40 | 8.74 | 8.40 | 8.40 | 8.40 | |
| Digestible threonine | 7.20 | 7.02 | 7.20 | | 7.20 | 7.20 | 6.83 | 7.20 | 7.20 | 7.20 | |
| Digestible tryptophane | 1.80 | 1.81 | 1.80 | | 1.80 | 1.80 | 1.71 | 1.80 | 1.80 | 1.80 | |
| Digestible arginine | 11.60 | 11.55 | 11.60 | | 11.60 | 11.60 | 11.53 | 11.60 | 11.60 | 11.60 | |
| Digestible valine | 8.40 | 7.96 | 8.40 | | 8.40 | 8.40 | 7.34 | 8.40 | 8.40 | 8.40 | |
| Linoleic acid C 18:2 | 30.00 | 28.18 | 30.00 | | 30.00 | 30.00 | 22.66 | 26.84 | 29.96 | 30.00 | |
| Control = Conventional corn-wheat based broiler diet  L-low = 5% of the daily dry matter feed intake is replaced by live black soldier fly larvae  MO-low = 5% of the daily dry matter feed intake is replaced by a mix of black soldier fly larvae meal & oil  M-low = the same amount of black soldier fly larvae meal is used as in diet MO-low  O-low = the same amount of black soldier fly larvae oil is used as in diet MO-low | | | | L-high = 10% of the daily dry matter feed intake is replaced by live black soldier fly larvae  MO-high = 10% of the daily dry matter feed intake is replaced by a mix of black soldier fly larvae meal & oil  M-high = the same amount of black soldier fly larvae meal is used as in diet MO-high  O-high = the same amount of black soldier fly larvae oil is used as in diet MO-high | | | | | | |  |

**Table S 3**: Nutrient composition of pelleted finisher feed per experimental treatment

| **Calculated nutrients**  **(g/kg as-fed, unless declared otherwise)** | **Control** | **L-**  **low** | **MO-**  **low** | | **M-**  **low** | **O-**  **low** | **L-**  **high** | **MO-**  **high** | **M-**  **high** | **O-**  **high** | |
| --- | --- | --- | --- | --- | --- | --- | --- | --- | --- | --- | --- |
| Dry matter | 886.89 | 883.80 | 887.24 | | 886.58 | 886.02 | 880.84 | 888.01 | 887.80 | 886.66 | |
| Crude protein | 177.91 | 170.93 | 180.18 | | 175.34 | 169.37 | 167.22 | 186.26 | 186.22 | 174.05 | |
| Crude fat | 109.91 | 85.35 | 102.42 | | 98.19 | 102.97 | 61.54 | 98.18 | 97.65 | 107.20 | |
| Crude fiber | 29.61 | 29.91 | 31.75 | | 31.48 | 29.11 | 30.53 | 34.14 | 34.15 | 29.38 | |
| Ash | 55.05 | 55.00 | 54.53 | | 53.78 | 53.73 | 55.93 | 54.60 | 54.60 | 54.47 | |
| Cl | 2.00 | 1.97 | 2.00 | | 2.00 | 2.00 | 2.03 | 2.00 | 2.00 | 2.00 | |
| K | 7.56 | 7.03 | 7.09 | | 6.71 | 6.88 | 6.78 | 6.93 | 6.93 | 7.25 | |
| Mg | 1.44 | 1.40 | 1.45 | | 1.40 | 1.36 | 1.39 | 1.49 | 1.49 | 1.41 | |
| Ca | 7.25 | 7.37 | 7.25 | | 7.25 | 7.25 | 7.50 | 7.24 | 7.25 | 7.25 | |
| Na | 1.60 | 1.59 | 1.60 | | 1.60 | 1.60 | 1.70 | 1.60 | 1.60 | 1.60 | |
| P | 5.66 | 5.86 | 5.93 | | 5.90 | 5.60 | 6.10 | 6.24 | 6.24 | 5.63 | |
| Retainable phosphorus (rP) | 3.22 | 3.41 | 3.22 | | 3.22 | 3.22 | 3.60 | 3.22 | 3.22 | 3.22 | |
| Ca : rP | 2.25 | 2.16 | 2.25 | | 2.25 | 2.25 | 2.09 | 2.25 | 2.25 | 2.25 | |
| Metabolizable energy (MJ) | 12.77 | 12.39 | 12.86 | | 12.81 | 12.85 | 11.96 | 12.94 | 12.82 | 12.90 | |
| Digestible lysine | 10.00 | 9.70 | 10.00 | | 10.00 | 10.00 | 9.36 | 10.00 | 10.00 | 10.00 | |
| Digestible methionine | 5.35 | 5.61 | 5.49 | | 5.58 | 5.48 | 5.77 | 5.57 | 5.59 | 5.39 | |
| Digestible cysteine | 2.50 | 2.40 | 2.34 | | 2.25 | 2.35 | 2.36 | 2.24 | 2.24 | 2.43 | |
| Digestible methionine + cysteine | 7.80 | 7.98 | 7.80 | | 7.80 | 7.80 | 8.12 | 7.80 | 7.80 | 7.80 | |
| Digestible threonine | 6.60 | 6.43 | 6.60 | | 6.60 | 6.60 | 6.23 | 6.60 | 6.60 | 6.60 | |
| Digestible tryptophane | 1.80 | 1.75 | 1.80 | | 1.80 | 1.80 | 1.59 | 1.80 | 1.80 | 1.80 | |
| Digestible arginine | 10.70 | 10.60 | 10.70 | | 10.70 | 10.70 | 10.47 | 10.70 | 10.70 | 10.70 | |
| Digestible valine | 7.70 | 7.26 | 7.80 | | 7.70 | 7.70 | 6.56 | 7.70 | 7.70 | 7.70 | |
| Linoleic acid C 18:2 | 30.00 | 28.17 | 30.00 | | 30.00 | 30.00 | 26.15 | 30.00 | 30.00 | 30.00 | |
| Control = Conventional corn-wheat based broiler diet  L-low = 5% of the daily dry matter feed intake is replaced by live black soldier fly larvae  MO-low = 5% of the daily dry matter feed intake is replaced by a mix of black soldier fly larvae meal & oil  M-low = the same amount of black soldier fly larvae meal is used as in diet MO-low  O-low = the same amount of black soldier fly larvae oil is used as in diet MO-low | | | | L-high = 10% of the daily dry matter feed intake is replaced by live black soldier fly larvae  MO-high = 10% of the daily dry matter feed intake is replaced by a mix of black soldier fly larvae meal & oil  M-high = the same amount of black soldier fly larvae meal is used as in diet MO-high  O-high = the same amount of black soldier fly larvae oil is used as in diet MO-high | | | | | | |  |

**Table S 4**: Analyzed proximate, mineral and amino acid composition (% w/w) of main feed ingredients

| **Nutritional values** | **Wheat** | **Corn** | **Soybean meal** | **Sunflower seed meal** | **Palm oil** | **Soybean oil** | |
| --- | --- | --- | --- | --- | --- | --- | --- |
| Moisture (4h, 103°C) | 13.80 | 12.80 | 11.80 | 9.00 |  |  | |
| Crude ash | 1.90 | 1.30 | 6.80 | 6.50 |  |  | |
| Crude protein (Nx6.25) | 11.50 | 7.50 | 45.40 | 33.40 |  |  | |
| Total fat | 1.90 | 3.70 | 2.20 | 1.90 | 98.30 | 99.50 | |
| Crude fibre | 2.50 | 1.60 | 3.90 | 16.80 |  |  | |
| N-free substances (calculated) | 68.40 | 73.10 | 29.90 | 32.40 |  |  | |
| **Minerals** |  |  |  |  |  |  | |
| Sodium | 0.03 | <0.01 | <0.01 | 0.01 | <0.01 | <0.01 | |
| Potassium | 0.43 | 0.32 | 2.18 | 1.43 | <0.01 | <0.01 | |
| Calcium | 0.08 | 0.01 | 0.30 | 0.42 | <0.01 | 0.01 | |
| Phosphorus | 0.28 | 0.21 | 0.61 | 1.02 | <0.01 | 0.02 | |
| Chloride | 0.10 | <0.10 | <0.10 | 0.11 |  |  | |
| **Amino acids** |  |  |  |  |  |  | |
| Lysine | 0.32 | 0.25 | 2.87 | 1.32 |  |  | |
| Methionine | 0.16 | 0.17 | 0.68 | 0.81 |  |  | |
| Cysteine | 0.24 | 0.17 | 0.69 | 0.56 |  |  | |
| Asparaginic acid | 0.58 | 0.51 | 5.45 | 3.15 |  |  | |
| Threonine | 0.31 | 0.26 | 1.84 | 1.29 |  |  | |
| Serine | 0.52 | 0.35 | 2.35 | 1.45 |  |  | |
| Glutamic acid | 3.44 | 1.45 | 8.78 | 6.89 |  |  | |
| Proline | 1.06 | 0.61 | 2.16 | 1.38 |  |  | |
| Glycine | 0.47 | 0.31 | 2.00 | 2.08 |  |  | |
| Alanine | 0.39 | 0.55 | 2.03 | 1.52 |  |  | |
| Valine | 0.48 | 0.37 | 2.26 | 1.76 |  |  | |
| Isoleucine | 0.38 | 0.27 | 2.21 | 1.43 |  |  | |
| Leucine | 0.75 | 0.89 | 3.60 | 2.24 |  |  | |
| Tyrosine | 0.25 | 0.23 | 1.41 | 0.64 |  |  | |
| Phenylalanine | 0.48 | 0.34 | 2.22 | 1.45 |  |  | |
| Histidine | 0.26 | 0.21 | 1.21 | 0.89 |  |  | |
| Arginine | 0.51 | 0.35 | 3.30 | 2.66 |  |  | |
| Tryptophan | 0.12 | 0.05 | 0.56 | 0.46 |  |  | |
| Sum amino acids | 10.70 | 7.34 | 45.60 | 32.00 |  |  | |
| If a table cell is empty, it means that the corresponding value was not measured. The symbol "<". in the result column means that the substance concerned was not quantifiable as it was below the limit of detection. | | | | | | |  |

**Table S 5**: Analyzed fatty acid composition expressed in % of total fatty acids of main feed ingredients

| **Fatty acids** | **Wheat** | **Corn** | **Soybean meal** | **Sunflower seed meal** | **Palm oil** | **Soybean oil** |
| --- | --- | --- | --- | --- | --- | --- |
| Octanoic acid | 0.1 | <0.1 | <0.1 | <0.1 | <0.1 | <0.1 |
| Capric acid | 0.1 | <0.1 | <0.1 | <0.1 | <0.1 | <0.1 |
| Lauric acid | 1.9 | 0.2 | <0.1 | 0.3 | 0.2 | <0.1 |
| Myristic acid | 0.8 | 0.1 | 0.1 | 0.3 | 1.0 | <0.1 |
| Pentadecanoic acid | 0.1 | <0.1 | <0.1 | 0.1 | <0.1 | <0.1 |
| Palmitic acid | 18.0 | 12.8 | 17.5 | 13.5 | 41.0 | 10.0 |
| Palmitoleinic acid | 0.2 | 0.2 | <0.1 | 0.2 | 0.2 | <0.1 |
| Margaric acid | 0.1 | <0.1 | 0.1 | 0.2 | 0.1 | 0.1 |
| Stearic acid | 1.1 | 2.3 | 3.5 | 4.2 | 4.2 | 4.6 |
| Octadecenoic acid trans-isomers | <0.1 | <0.1 | 0.1 | 0.1 | <0.1 | <0.1 |
| Oleic acid | 12.7 | 28.2 | 14.8 | 20.9 | 40.5 | 21.1 |
| Cis-vaccenic acid | 1.0 | 0.7 | 1.7 | 0.8 | 0.8 | 1.3 |
| Octadecadienoic acid trans- isomers | <0.1 | <0.1 | 0.2 | 0.2 | <0.1 | <0.1 |
| Linolic acid | 57.5 | 52.0 | 53.4 | 55.5 | 10.8 | 53.9 |
| Alpha-linolenic acid | 4.5 | 1.9 | 6.8 | 1.7 | 0.3 | 7.4 |
| Arachic acid | 0.2 | 0.5 | 0.3 | 0.4 | 0.4 | 0.4 |
| Eicosenoic acid | 0.7 | 0.4 | 0.2 | 0.2 | 0.2 | 0.2 |
| Eicosadienic acid | 0.1 | <0.1 | <0.1 | <0.1 | <0.1 | <0.1 |
| Behenic acid | 0.2 | 0.2 | 0.4 | 0.7 | <0.1 | 0.4 |
| Docosenoic acid | 0.1 | <0.1 | <0.1 | <0.1 | <0.1 | <0.1 |
| Tricosanoic acid | <0.1 | <0.1 | <0.1 | 0.1 | <0.1 | <0.1 |
| Lignoceric acid | 0.2 | 0.3 | 0.3 | 0.4 | <0.1 | 0.2 |
| Sum saturated fatty acids | 22.8 | 16.4 | 22.2 | 20.2 | 46.9 | 15.7 |
| Sum monounsaturated fatty acids | 14.7 | 29.5 | 16.8 | 22.2 | 41.7 | 22.6 |
| Sum polyunsaturated fatty acids | 62.1 | 53.9 | 60.4 | 57.4 | 11.1 | 61.3 |
| If a table cell is empty, it means that the corresponding value was not measured. The symbol "<" in the result column means that the substance concerned was not quantifiable as it was below the limit of detection. The sum calculations were done without taking into account values below the detection limit. | | | | | | |

**Table S 6**: Analyzed proximate and mineral composition (% w/w) of pelleted starter feed

| **Nutritional values** | **Control** | **L-low** | **MO-low** | **M-low** | **O-low** | **L-high** | **MO-high** | **M-high** | **O-high** |
| --- | --- | --- | --- | --- | --- | --- | --- | --- | --- |
| Moisture (4h, 103°C) | 10.90 | 10.90 | 10.50 | 10.50 | 10.80 | 11.10 | 10.40 | 10.60 | 10.70 |
| Crude ash | 5.60 | 5.60 | 5.40 | 5.50 | 5.30 | 5.60 | 5.50 | 5.50 | 5.50 |
| Crude protein (Nx6.25) | 19.20 | 19.40 | 20.00 | 20.10 | 19.70 | 18.70 | 20.30 | 20.00 | 19.50 |
| Crude fat | 8.00 | 5.50 | 7.20 | 7.20 | 7.40 | 2.80 | 6.70 | 6.70 | 7.80 |
| Crude fibre | 2.80 | 2.60 | 3.00 | 2.90 | 2.80 | 2.80 | 3.30 | 3.20 | 2.90 |
| N-free substances (calculated) | 53.50 | 56.00 | 53.90 | 53.80 | 54.00 | 59.00 | 53.80 | 54.00 | 53.60 |
| **Minerals** |  |  |  |  |  |  |  |  |  |
| Sodium | 0.15 | 0.16 | 0.15 | 0.14 | 0.15 | 0.14 | 0.14 | 0.17 | 0.15 |
| Potassium | 0.83 | 0.81 | 0.76 | 0.80 | 0.81 | 0.77 | 0.76 | 0.77 | 0.81 |
| Calcium | 0.87 | 0.93 | 0.88 | 0.85 | 0.85 | 0.87 | 0.82 | 0.96 | 0.87 |
| Phosphorus | 0.66 | 0.69 | 0.67 | 0.68 | 0.65 | 0.69 | 0.69 | 0.73 | 0.66 |
| Chloride | 0.20 | 0.20 | 0.19 | 0.21 | 0.21 | 0.22 | 0.20 | 0.21 | 0.21 |
| Control = Conventional corn-wheat based broiler diet  L-low = 5% of the daily dry matter feed intake is replaced by live black soldier fly larvae  MO-low = 5% of the daily dry matter feed intake is replaced by a mix of black soldier fly larvae meal & oil  M-low = the same amount of black soldier fly larvae meal is used as in diet MO-low  O-low = the same amount of black soldier fly larvae oil is used as in diet MO-low | | | | L-high = 10% of the daily dry matter feed intake is replaced by live black soldier fly larvae  MO-high = 10% of the daily dry matter feed intake is replaced by a mix of black soldier fly larvae meal & oil  M-high = the same amount of black soldier fly larvae meal is used as in diet MO-high  O-high = the same amount of black soldier fly larvae oil is used as in diet MO-high | | | | | |

**Table S 7**: Analyzed amino acid composition (% w/w) of pelleted starter feed

| **Amino acids** | **Control** | **L-low** | **MO-low** | **M-low** | **O-low** | **L-high** | **MO-high** | **M-high** | **O-high** |
| --- | --- | --- | --- | --- | --- | --- | --- | --- | --- |
| Lysine | 1.33 | 1.30 | 1.36 | 1.37 | 1.33 | 1.34 | 1.46 | 1.45 | 1.37 |
| Methionine | 0.62 | 0.63 | 0.67 | 0.67 | 0.64 | 0.65 | 0.71 | 0.76 | 0.59 |
| Cysteine | 0.32 | 0.32 | 0.31 | 0.31 | 0.32 | 0.29 | 0.30 | 0.28 | 0.31 |
| Asparaginic acid | 1.82 | 1.74 | 1.73 | 1.77 | 1.78 | 1.62 | 1.77 | 1.73 | 1.79 |
| Threonine | 0.86 | 0.83 | 0.87 | 0.88 | 0.85 | 0.80 | 0.87 | 0.87 | 0.83 |
| Serine | 0.89 | 0.87 | 0.85 | 0.88 | 0.87 | 0.84 | 0.84 | 0.83 | 0.87 |
| Glutamic acid | 3.66 | 3.58 | 3.47 | 3.54 | 3.62 | 3.46 | 3.44 | 3.41 | 3.58 |
| Proline | 1.09 | 1.10 | 1.16 | 1.16 | 1.11 | 1.13 | 1.13 | 1.14 | 1.14 |
| Glycine | 0.78 | 0.76 | 0.79 | 0.80 | 0.78 | 0.73 | 0.84 | 0.82 | 0.78 |
| Alanine | 0.89 | 0.88 | 0.91 | 0.93 | 0.88 | 0.81 | 0.96 | 0.94 | 0.85 |
| Valine | 1.03 | 0.99 | 1.03 | 1.05 | 1.00 | 0.93 | 1.05 | 1.04 | 1.02 |
| Isoleucine | 0.85 | 0.84 | 0.84 | 0.85 | 0.85 | 0.78 | 0.82 | 0.81 | 0.81 |
| Leucine | 1.50 | 1.48 | 1.44 | 1.46 | 1.47 | 1.39 | 1.45 | 1.42 | 1.44 |
| Tyrosine | 0.62 | 0.61 | 0.68 | 0.69 | 0.65 | 0.55 | 0.66 | 0.70 | 0.58 |
| Phenylalanine | 0.90 | 0.87 | 0.90 | 0.91 | 0.97 | 0.85 | 0.86 | 0.87 | 0.89 |
| Histidine | 0.48 | 0.46 | 0.47 | 0.48 | 0.47 | 0.38 | 0.41 | 0.42 | 0.39 |
| Arginine | 1.33 | 1.34 | 1.32 | 1.35 | 1.36 | 1.30 | 1.34 | 1.32 | 1.26 |
| Tryptophan | 0.21 | 0.20 | 0.21 | 0.22 | 0.20 | 0.19 | 0.22 | 0.22 | 0.20 |
| Sum amino acids | 19.20 | 18.80 | 19.00 | 19.30 | 19.20 | 18.00 | 19.10 | 19.00 | 18.70 |
| Control = Conventional corn-wheat based broiler diet  L-low = 5% of the daily dry matter feed intake is replaced by live black soldier fly larvae  MO-low = 5% of the daily dry matter feed intake is replaced by a mix of black soldier fly larvae meal & oil  M-low = the same amount of black soldier fly larvae meal is used as in diet MO-low  O-low = the same amount of black soldier fly larvae oil is used as in diet MO-low | | | | L-high = 10% of the daily dry matter feed intake is replaced by live black soldier fly larvae  MO-high = 10% of the daily dry matter feed intake is replaced by a mix of black soldier fly larvae meal & oil  M-high = the same amount of black soldier fly larvae meal is used as in diet MO-high  O-high = the same amount of black soldier fly larvae oil is used as in diet MO-high | | | | | |

**Table S 8**: Analyzed fatty acid composition (mg/kg w/w) of pelleted starter feed

| **Fatty acids** | **Control** | **L-low** | **MO-low** | | **M-low** | **O-low** | **L-high** | **MO-high** | **M-high** | **O-high** |
| --- | --- | --- | --- | --- | --- | --- | --- | --- | --- | --- |
| Capric acid | <50.0 | <50.0 | 179.0 | | <50.0 | 138.0 | <50.0 | 342.0 | 77.9 | 274.0 |
| Lauric acid | 95.0 | 136.0 | 7250.0 | | 1530.0 | 5480.0 | 137.0 | 14100.0 | 3010.0 | 11300.0 |
| Myristic acid | 82.3 | 81.2 | 1740.0 | | 413.0 | 1330.0 | 63.1 | 3320.0 | 760.0 | 2670.0 |
| Myristoleic acid | <50.0 | <50.0 | <50.0 | | <50.0 | <50.0 | <50.0 | 83.1 | <50.0 | 70.9 |
| Palmitic acid | 7410.0 | 5460.0 | 8140.0 | | 6910.0 | 8090.0 | 3270.0 | 8400.0 | 6960.0 | 8950.0 |
| Hexadecanoic acid trans-isomers | <50.0 | <50.0 | <50.0 | | <50.0 | 59.6 | <50.0 | <50.0 | <50.0 | <50.0 |
| Palmitoleinic acid | 70.2 | 51.0 | 592.0 | | 173.0 | 453.0 | <50.0 | 1090.0 | 300.0 | 869.0 |
| Margaric acid | 68.0 | <50.0 | 65.4 | | 63.1 | 67.9 | <50.0 | 71.5 | 60.7 | 82.0 |
| Stearic acid | 2500.0 | 1580.0 | 2040.0 | | 2160.0 | 2250.0 | 572.0 | 1470.0 | 2000.0 | 2000.0 |
| Oleic acid | 14000.0 | 9950.0 | 11700.0 | | 12300.0 | 12600.0 | 5450.0 | 8700.0 | 11600.0 | 11300.0 |
| Petroselinic acid | <50.0 | <50.0 | <50.0 | | <50.0 | <50.0 | 8220.0 | <50.0 | <50.0 | <50.0 |
| Cis-vaccenic acid | 735.0 | 493.0 | 555.0 | | 627.0 | 632.0 | 209.0 | 331.0 | 551.0 | 531.0 |
| Linolic acid | 34300.0 | 23900.0 | 26200.0 | | 29200.0 | 29200.0 | 12300.0 | 16500.0 | 26700.0 | 24400.0 |
| Alpha-linolenic acid | 3820.0 | 2350.0 | 2540.0 | | 3130.0 | 3030.0 | 759.0 | 1120.0 | 2720.0 | 2260.0 |
| Arachic acid | 266.0 | 188.0 | 207.0 | | 229.0 | 225.0 | 97.4 | 126.0 | 209.0 | 187.0 |
| Eicosenoic acid | 181.0 | 141.0 | 161.0 | | 162.0 | 166.0 | 83.2 | 113.0 | 151.0 | 149.0 |
| Behenic acid | 243.0 | 158.0 | 159.0 | | 197.0 | 186.0 | 64.1 | 63.5 | 176.0 | 138.0 |
| Lignoceric acid | 119.0 | 87.9 | 95.1 | | 108.0 | 102.0 | 60.4 | 59.8 | 90.5 | 82.7 |
| Sum saturated fatty acids | 10800.0 | 7690.0 | 19900.0 | | 11600.0 | 17900.0 | 4260.0 | 28000.0 | 13300.0 | 25700.0 |
| Sum monounsaturated fatty acids | 15000.0 | 10600.0 | 13000.0 | | 13300.0 | 13900.0 | 14000.0 | 10300.0 | 12600.0 | 12900.0 |
| Sum polyunsaturated fatty acids | 38100.0 | 26300.0 | 28700.0 | | 32300.0 | 32200.0 | 13100.0 | 17600.0 | 29400.0 | 26700.0 |
| Total sum of fatty acids | 63900.0 | 44600.0 | 61600.0 | | 57200.0 | 64000.0 | 31300.0 | 55900.0 | 55400.0 | 65300.0 |
| The symbol "<" in the result column means that the substance concerned was not quantifiable as it was below the limit of detection. The sum calculations were done without taking into account values below the detection limit.  Control = Conventional corn-wheat based broiler diet  L-low = 5% of the daily dry matter feed intake is replaced by live black soldier fly larvae  MO-low = 5% of the daily dry matter feed intake is replaced by a mix of black soldier fly larvae meal & oil  M-low = the same amount of black soldier fly larvae meal is used as in diet MO-low  O-low = the same amount of black soldier fly larvae oil is used as in diet MO-low | | | | L-high = 10% of the daily dry matter feed intake is replaced by live black soldier fly larvae  MO-high = 10% of the daily dry matter feed intake is replaced by a mix of black soldier fly larvae meal & oil  M-high = the same amount of black soldier fly larvae meal is used as in diet MO-high  O-high = the same amount of black soldier fly larvae oil is used as in diet MO-high | | | | | | |

**Table S 9**: Analyzed proximate and mineral composition (% w/w) of pelleted grower feed

| **Nutritional values** | **Control** | **L-low** | **MO-low** | **M-low** | **O-low** | **L-high** | **MO-high** | **M-high** | **O-high** |
| --- | --- | --- | --- | --- | --- | --- | --- | --- | --- |
| Moisture (4h, 103°C) | 10.90 | 11.50 | 11.00 | 11.00 | 10.80 | 11.80 | 11.10 | 11.10 | 10.80 |
| Crude ash | 5.10 | 5.00 | 5.10 | 5.10 | 5.20 | 5.20 | 4.90 | 5.20 | 5.10 |
| Crude protein (Nx6.25) | 18.30 | 18.20 | 19.00 | 18.50 | 18.30 | 17.30 | 19.30 | 19.40 | 18.50 |
| Crude fat | 9.90 | 7.40 | 9.40 | 9.30 | 10.00 | 5.10 | 8.20 | 8.90 | 9.60 |
| Crude fibre | 3.00 | 3.40 | 3.20 | 3.30 | 3.00 | 3.00 | 3.40 | 3.00 | 2.80 |
| N-free substances (calculated) | 52.80 | 54.50 | 52.30 | 52.80 | 52.70 | 57.60 | 53.10 | 52.40 | 53.20 |
| **Minerals** |  |  |  |  |  |  |  |  |  |
| Sodium | 0.15 | 0.18 | 0.16 | 0.16 | 0.15 | 0.16 | 0.16 | 0.16 | 0.16 |
| Potassium | 0.78 | 0.72 | 0.74 | 0.74 | 0.76 | 0.71 | 0.70 | 0.69 | 0.75 |
| Calcium | 0.84 | 0.90 | 0.87 | 0.85 | 0.82 | 0.88 | 0.87 | 0.86 | 0.85 |
| Phosphorus | 0.63 | 0.65 | 0.66 | 0.66 | 0.62 | 0.68 | 0.68 | 0.67 | 0.62 |
| Chloride | 0.21 | 0.22 | 0.26 | 0.21 | 0.20 | 0.21 | 0.20 | 0.18 | 0.20 |
| Control = Conventional corn-wheat based broiler diet  L-low = 5% of the daily dry matter feed intake is replaced by live black soldier fly larvae  MO-low = 5% of the daily dry matter feed intake is replaced by a mix of black soldier fly larvae meal & oil  M-low = the same amount of black soldier fly larvae meal is used as in diet MO-low  O-low = the same amount of black soldier fly larvae oil is used as in diet MO-low | | | | L-high = 10% of the daily dry matter feed intake is replaced by live black soldier fly larvae  MO-high = 10% of the daily dry matter feed intake is replaced by a mix of black soldier fly larvae meal & oil  M-high = the same amount of black soldier fly larvae meal is used as in diet MO-high  O-high = the same amount of black soldier fly larvae oil is used as in diet MO-high | | | | | |

**Table S 10**: Analyzed amino acid composition (% w/w) of pelleted grower feed

| **Amino acids** | **Control** | **L-low** | **MO-low** | **M-low** | **O-low** | **L-high** | **MO-high** | **M-high** | **O-high** |
| --- | --- | --- | --- | --- | --- | --- | --- | --- | --- |
| Lysine | 1.14 | 1.13 | 1.19 | 1.18 | 1.18 | 1.11 | 1.20 | 1.18 | 1.16 |
| Methionine sulfone | 0.58 | 0.62 | 0.56 | 0.57 | 0.55 | 0.63 | 0.65 | 0.68 | 0.56 |
| Cysteine | 0.33 | 0.31 | 0.33 | 0.31 | 0.30 | 0.26 | 0.31 | 0.27 | 0.30 |
| Asparaginic acid | 1.56 | 1.38 | 1.49 | 1.46 | 1.55 | 1.30 | 1.53 | 1.46 | 1.55 |
| Threonine | 0.78 | 0.73 | 0.75 | 0.74 | 0.74 | 0.73 | 0.81 | 0.79 | 0.77 |
| Serine | 0.80 | 0.72 | 0.77 | 0.76 | 0.79 | 0.70 | 0.80 | 0.75 | 0.78 |
| Glutamic acid | 3.62 | 3.35 | 3.41 | 3.38 | 3.51 | 3.25 | 3.47 | 3.42 | 3.59 |
| Proline | 1.09 | 1.10 | 1.05 | 1.05 | 1.12 | 1.04 | 1.00 | 1.14 | 1.00 |
| Glycine | 0.72 | 0.67 | 0.73 | 0.74 | 0.71 | 0.64 | 0.79 | 0.78 | 0.73 |
| Alanine | 0.76 | 0.67 | 0.78 | 0.79 | 0.75 | 0.65 | 0.89 | 0.84 | 0.77 |
| Valine | 0.95 | 0.87 | 0.93 | 0.92 | 0.97 | 0.83 | 0.96 | 0.94 | 0.97 |
| Isoleucine | 0.80 | 0.70 | 0.76 | 0.77 | 0.79 | 0.66 | 0.79 | 0.76 | 0.80 |
| Leucine | 1.33 | 1.18 | 1.28 | 1.27 | 1.33 | 1.14 | 1.33 | 1.28 | 1.30 |
| Tyrosine | 0.55 | 0.49 | 0.55 | 0.56 | 0.56 | 0.46 | 0.49 | 0.53 | 0.46 |
| Phenylalanine | 0.82 | 0.73 | 0.77 | 0.78 | 0.86 | 0.75 | 0.70 | 0.72 | 0.74 |
| Histidine | 0.49 | 0.36 | 0.34 | 0.32 | 0.35 | 0.35 | 0.44 | 0.42 | 0.42 |
| Arginine | 1.21 | 1.13 | 1.11 | 1.10 | 1.17 | 1.16 | 1.18 | 1.15 | 1.12 |
| Tryptophan | 0.20 | 0.20 | 0.20 | 0.21 | 0.22 | 0.18 | 0.22 | 0.22 | 0.21 |
| Sum amino acids | 17.70 | 16.30 | 17.00 | 16.90 | 17.50 | 15.80 | 17.60 | 17.30 | 17.20 |
| Control = Conventional corn-wheat based broiler diet  L-low = 5% of the daily dry matter feed intake is replaced by live black soldier fly larvae  MO-low = 5% of the daily dry matter feed intake is replaced by a mix of black soldier fly larvae meal & oil  M-low = the same amount of black soldier fly larvae meal is used as in diet MO-low  O-low = the same amount of black soldier fly larvae oil is used as in diet MO-low | | | | L-high = 10% of the daily dry matter feed intake is replaced by live black soldier fly larvae  MO-high = 10% of the daily dry matter feed intake is replaced by a mix of black soldier fly larvae meal & oil  M-high = the same amount of black soldier fly larvae meal is used as in diet MO-high  O-high = the same amount of black soldier fly larvae oil is used as in diet MO-high | | | | | |

**Table S 11**: Analyzed fatty acid composition (mg/kg w/w) of pelleted grower feed

| **Fatty acids** | **Control** | **L-low** | **MO-low** | | **M-low** | **O-low** | **L-high** | **MO-high** | **M-high** | **O-high** |
| --- | --- | --- | --- | --- | --- | --- | --- | --- | --- | --- |
| Capric acid | <50.0 | <50.0 | 196.0 | | 55.3 | 152.0 | <50.0 | 363.0 | 94.4 | 316.0 |
| Lauric acid | 208.0 | 206.0 | 7580.0 | | 1720.0 | 5710.0 | 184.0 | 14600.0 | 3310.0 | 12600.0 |
| Myristic acid | 579.0 | 383.0 | 2060.0 | | 834.0 | 1700.0 | 200.0 | 3530.0 | 1130.0 | 3200.0 |
| Myristoleic acid | <50.0 | <50.0 | <50.0 | | <50.0 | <50.0 | <50.0 | 85.9 | <50.0 | 71.9 |
| Pentadecanoic acid | <50.0 | <50.0 | <50.0 | | <50.0 | <50.0 | <50.0 | <50.0 | <50.0 | 59.8 |
| Palmitic acid | 26500.0 | 17600.0 | 19200.0 | | 22700.0 | 21800.0 | 9120.0 | 13700.0 | 20000.0 | 20200.0 |
| Palmitoleinic acid | 145.0 | 108.0 | 653.0 | | 254.0 | 522.0 | 72.1 | 1140.0 | 376.0 | 1000.0 |
| Margaric acid | 97.5 | 77.0 | 99.7 | | 94.2 | 96.8 | 56.3 | 97.7 | 96.4 | 102.0 |
| Stearic acid | 3460.0 | 2670.0 | 2970.0 | | 3180.0 | 3060.0 | 1620.0 | 2390.0 | 3100.0 | 3130.0 |
| Octadecenoic acid trans-isomers | <50.0 | <50.0 | <50.0 | | <50.0 | 51.4 | <50.0 | <50.0 | <50.0 | <50.0 |
| Oleic acid | 26200.0 | 19000.0 | 19500.0 | | 22900.0 | 21500.0 | 11100.0 | 13600.0 | 21200.0 | 19800.0 |
| Cis-vaccenic acid | 822.0 | 685.0 | 710.0 | | 752.0 | 730.0 | 464.0 | 553.0 | 741.0 | 745.0 |
| Octadecadienoic acid | 87.9 | 67.7 | 74.4 | | 67.3 | 76.0 | 54.8 | 67.2 | 70.9 | 72.6 |
| Linolic acid | 24800.0 | 24200.0 | 25300.0 | | 24800.0 | 24300.0 | 19200.0 | 22200.0 | 26200.0 | 26600.0 |
| Octadecatetrienic acid | <50.0 | <50.0 | <50.0 | | <50.0 | 56.4 | <50.0 | <50.0 | <50.0 | <50.0 |
| Alpha-linolenic acid | 2200.0 | 2280.0 | 2350.0 | | 2280.0 | 2190.0 | 1750.0 | 1950.0 | 2480.0 | 2420.0 |
| Arachic acid | 318.0 | 257.0 | 261.0 | | 289.0 | 271.0 | 168.0 | 186.0 | 278.0 | 262.0 |
| Eicosenoic acid | 199.0 | 174.0 | 306.0 | | 207.0 | 277.0 | 135.0 | 395.0 | 231.0 | 415.0 |
| Eicosapentaenic acid | <50.0 | <50.0 | 75.4 | | <50.0 | 55.0 | <50.0 | 89.1 | <50.0 | 74.7 |
| Behenic acid | 166.0 | 160.0 | 151.0 | | 158.0 | 149.0 | 118.0 | 112.0 | 168.0 | 150.0 |
| Lignoceric acid | 112.0 | 103.0 | 94.5 | | 107.0 | 99.7 | 81.1 | 65.3 | 105.0 | 95.4 |
| Sum saturated fatty acids | 31400.0 | 21500.0 | 32600.0 | | 29100.0 | 33000.0 | 11500.0 | 35000.0 | 28300.0 | 40100.0 |
| Sum monounsaturated fatty acids | 27400.0 | 20000.0 | 21200.0 | | 24100.0 | 23100.0 | 11800.0 | 15800.0 | 22500.0 | 22000.0 |
| Sum polyunsaturated fatty acids | 27100.0 | 26500.0 | 27800.0 | | 27100.0 | 26700.0 | 21000.0 | 24300.0 | 28800.0 | 29200.0 |
| Total sum of fatty acids | 85900.0 | 68000.0 | 81600.0 | | 80400.0 | 82800.0 | 44300.0 | 75100.0 | 79600.0 | 91300.0 |
| The symbol "<" in the result column means that the substance concerned was not quantifiable as it was below the limit of detection. The sum calculations were done without taking into account values below the detection limit.  Control = Conventional corn-wheat based broiler diet  L-low = 5% of the daily dry matter feed intake is replaced by live black soldier fly larvae  MO-low = 5% of the daily dry matter feed intake is replaced by a mix of black soldier fly larvae meal & oil  M-low = the same amount of black soldier fly larvae meal is used as in diet MO-low | | | | O-low = the same amount of black soldier fly larvae oil is used as in diet MO-low  L-high = 10% of the daily dry matter feed intake is replaced by live black soldier fly larvae  MO-high = 10% of the daily dry matter feed intake is replaced by a mix of black soldier fly larvae meal & oil, M-high = the same amount of black soldier fly larvae meal is used as in diet MO-high  O-high = the same amount of black soldier fly larvae oil is used as in diet MO-high | | | | | | |

**Table S 12**: Analyzed proximate and mineral composition (% w/w) of pelleted finisher feed

| **Nutritional values** | **Control** | **L-low** | **MO-low** | **M-low** | **O-low** | **L-high** | **MO-high** | **M-high** | **O-high** |
| --- | --- | --- | --- | --- | --- | --- | --- | --- | --- |
| Moisture (4h, 103°C) | 11.00 | 11.20 | 10.80 | 11.20 | 11.10 | 11.70 | 11.00 | 11.10 | 10.90 |
| Crude ash | 4.80 | 4.60 | 4.80 | 4.60 | 4.80 | 4.70 | 4.80 | 4.60 | 4.60 |
| Crude protein (Nx6,25) | 18.20 | 17.00 | 17.90 | 17.90 | 17.20 | 17.10 | 19.00 | 19.10 | 17.90 |
| Crude fat | 11.00 | 8.90 | 10.90 | 10.00 | 10.70 | 6.10 | 9.90 | 9.80 | 10.50 |
| Crude fibre | 2.80 | 3.10 | 2.90 | 3.30 | 3.20 | 3.00 | 3.70 | 3.30 | 2.90 |
| N-free substances (calculated) | 52.20 | 55.20 | 52.70 | 53.00 | 53.00 | 57.40 | 51.60 | 52.10 | 53.20 |
| **Minerals** |  |  |  |  |  |  |  |  |  |
| Sodium | 0.14 | 0.15 | 0.15 | 0.16 | 0.14 | 0.14 | 0.14 | 0.14 | 0.15 |
| Potassium | 0.76 | 0.69 | 0.70 | 0.64 | 0.71 | 0.68 | 0.70 | 0.79 | 0.75 |
| Calcium | 0.68 | 0.70 | 0.71 | 0.73 | 0.63 | 0.68 | 0.69 | 0.67 | 0.70 |
| Phosphorus | 0.55 | 0.56 | 0.59 | 0.58 | 0.55 | 0.57 | 0.61 | 0.57 | 0.57 |
| Chloride | 0.20 | 0.20 | 0.20 | 0.21 | 0.20 | 0.22 | 0.19 | 0.20 | 0.20 |
| Control = Conventional corn-wheat based broiler diet  L-low = 5% of the daily dry matter feed intake is replaced by live black soldier fly larvae  MO-low = 5% of the daily dry matter feed intake is replaced by a mix of black soldier fly larvae meal & oil  M-low = the same amount of black soldier fly larvae meal is used as in diet MO-low  O-low = the same amount of black soldier fly larvae oil is used as in diet MO-low | | | | L-high = 10% of the daily dry matter feed intake is replaced by live black soldier fly larvae  MO-high = 10% of the daily dry matter feed intake is replaced by a mix of black soldier fly larvae meal & oil  M-high = the same amount of black soldier fly larvae meal is used as in diet MO-high  O-high = the same amount of black soldier fly larvae oil is used as in diet MO-high | | | | | |

**Table S 13**: Analyzed amino acid composition (% w/w) of pelleted finisher feed

| **Amino acids** | **Control** | **L-low** | **MO-low** | **M-low** | **O-low** | **L-high** | **MO-high** | **M-high** | **O-high** |
| --- | --- | --- | --- | --- | --- | --- | --- | --- | --- |
| Lysine | 1.09 | 1.05 | 1.11 | 1.11 | 1.09 | 1.07 | 1.13 | 1.13 | 1.09 |
| Methionine | 0.53 | 0.57 | 0.56 | 0.59 | 0.54 | 0.57 | 0.53 | 0.60 | 0.52 |
| Cysteine | 0.30 | 0.28 | 0.28 | 0.26 | 0.28 | 0.29 | 0.29 | 0.28 | 0.29 |
| Asparaginic acid | 1.63 | 1.43 | 1.47 | 1.39 | 1.43 | 1.40 | 1.56 | 1.49 | 1.47 |
| Threonine | 0.75 | 0.73 | 0.75 | 0.75 | 0.73 | 0.69 | 0.76 | 0.76 | 0.74 |
| Serine | 0.85 | 0.77 | 0.77 | 0.74 | 0.76 | 0.73 | 0.82 | 0.79 | 0.77 |
| Glutamic acid | 3.77 | 3.55 | 3.50 | 3.42 | 3.56 | 3.60 | 3.58 | 3.47 | 3.57 |
| Proline | 1.17 | 1.10 | 1.13 | 1.09 | 1.10 | 1.10 | 1.22 | 1.23 | 1.12 |
| Glycine | 0.76 | 0.70 | 0.74 | 0.72 | 0.70 | 0.71 | 0.81 | 0.78 | 0.70 |
| Alanine | 0.79 | 0.73 | 0.77 | 0.75 | 0.71 | 0.72 | 0.88 | 0.85 | 0.72 |
| Valine | 0.88 | 0.84 | 0.85 | 0.85 | 0.85 | 0.77 | 0.91 | 0.88 | 0.87 |
| Isoleucine | 0.77 | 0.71 | 0.71 | 0.72 | 0.73 | 0.67 | 0.77 | 0.74 | 0.74 |
| Leucine | 1.37 | 1.24 | 1.25 | 1.20 | 1.21 | 1.23 | 1.39 | 1.32 | 1.26 |
| Tyrosine | 0.58 | 0.53 | 0.56 | 0.54 | 0.55 | 0.49 | 0.65 | 0.63 | 0.51 |
| Phenylalanine | 0.86 | 0.79 | 0.74 | 0.73 | 0.75 | 0.76 | 0.84 | 0.82 | 0.79 |
| Histidine | 0.47 | 0.45 | 0.44 | 0.40 | 0.42 | 0.40 | 0.45 | 0.44 | 0.41 |
| Arginine | 1.18 | 1.10 | 1.11 | 1.12 | 1.14 | 1.14 | 1.18 | 1.15 | 1.13 |
| Tryptophan | 0.20 | 0.19 | 0.22 | 0.22 | 0.21 | 0.19 | 0.22 | 0.22 | 0.21 |
| Sum amino acids | 18.00 | 16.80 | 17.00 | 16.60 | 16.80 | 16.50 | 18.00 | 17.60 | 16.90 |
| Control = Conventional corn-wheat based broiler diet  L-low = 5% of the daily dry matter feed intake is replaced by live black soldier fly larvae  MO-low = 5% of the daily dry matter feed intake is replaced by a mix of black soldier fly larvae meal & oil  M-low = the same amount of black soldier fly larvae meal is used as in diet MO-low  O-low = the same amount of black soldier fly larvae oil is used as in diet MO-low | | | | L-high = 10% of the daily dry matter feed intake is replaced by live black soldier fly larvae  MO-high = 10% of the daily dry matter feed intake is replaced by a mix of black soldier fly larvae meal & oil  M-high = the same amount of black soldier fly larvae meal is used as in diet MO-high  O-high = the same amount of black soldier fly larvae oil is used as in diet MO-high | | | | | |

**Table S 14**: Analyzed fatty acid composition (mg/kg w/w) of pelleted finisher feed

| **Fatty acids** | **Control** | **L-low** | **MO-low** | | **M-low** | **O-low** | **L-high** | **MO-high** | **M-high** | **O-high** |
| --- | --- | --- | --- | --- | --- | --- | --- | --- | --- | --- |
| Capric acid | <50.0 | <50.0 | 211.0 | | <50.0 | 161.0 | <50.0 | 359.0 | 81.6 | 300.0 |
| Lauric acid | 201.0 | 156.0 | 8200.0 | | 1620.0 | 6410.0 | 124.0 | 14900.0 | 3030.0 | 12100.0 |
| Myristic acid | 703.0 | 439.0 | 2300.0 | | 852.0 | 1940.0 | 226.0 | 3670.0 | 1150.0 | 3160.0 |
| Myristoleic acid | <50.0 | <50.0 | <50.0 | | <50.0 | <50.0 | <50.0 | 91.7 | <50.0 | 69.8 |
| Pentadecanoic acid | <50.0 | <50.0 | 51.9 | | <50.0 | <50.0 | <50.0 | 57.8 | <50.0 | 55.3 |
| Palmitic acid | 33700.0 | 21500.0 | 25100.0 | | 25400.0 | 26000.0 | 12000.0 | 16600.0 | 23900.0 | 23500.0 |
| Palmitoleinic acid | 156.0 | 110.0 | 666.0 | | 256.0 | 526.0 | 68.8 | 1210.0 | 353.0 | 934.0 |
| Margaric acid | 108.0 | 83.1 | 105.0 | | 99.6 | 97.1 | 60.8 | 105.0 | 89.4 | 108.0 |
| Stearic acid | 4000.0 | 2860.0 | 3430.0 | | 3310.0 | 3420.0 | 1980.0 | 2790.0 | 3250.0 | 3240.0 |
| Octadecenoic acid trans-isomers | <50.0 | <50.0 | 52.0 | | <50.0 | <50.0 | <50.0 | <50.0 | <50.0 | <50.0 |
| Oleic acid | 31700.0 | 21700.0 | 24200.0 | | 24900.0 | 24900.0 | 14000.0 | 16500.0 | 23800.0 | 22100.0 |
| Cis-vaccenic acid | 1080.0 | 802.0 | 912.0 | | 909.0 | 905.0 | 624.0 | 718.0 | 860.0 | 842.0 |
| Octadecadienoic acid | <50.0 | <50.0 | 96.0 | | <50.0 | <50.0 | 86.4 | <50.0 | <50.0 | <50.0 |
| Linolic acid | 25900.0 | 23800.0 | 27000.0 | | 25300.0 | 26300.0 | 23000.0 | 26300.0 | 25300.0 | 26000.0 |
| Alpha-linolenic acid | 2170.0 | 2130.0 | 2380.0 | | 2250.0 | 2310.0 | 2180.0 | 2420.0 | 2270.0 | 2270.0 |
| Arachic acid | 354.0 | 263.0 | 287.0 | | 295.0 | 299.0 | 197.0 | 218.0 | 284.0 | 265.0 |
| Eicosenoic acid | 211.0 | 173.0 | 194.0 | | 186.0 | 194.0 | 145.0 | 174.0 | 191.0 | 179.0 |
| Behenic acid | 155.0 | 135.0 | 146.0 | | 152.0 | 144.0 | 132.0 | 126.0 | 146.0 | 129.0 |
| Lignoceric acid | 100.0 | 74.6 | 90.5 | | 94.2 | 85.9 | 77.3 | 64.9 | 90.6 | 82.4 |
| Sum saturated fatty acids | 39300.0 | 25500.0 | 39900.0 | | 31800.0 | 38600.0 | 14800.0 | 38900.0 | 32000.0 | 42900.0 |
| Sum monounsaturated fatty acids | 33100.0 | 22800.0 | 26000.0 | | 26300.0 | 26500.0 | 14800.0 | 18700.0 | 25200.0 | 24100.0 |
| Sum polyunsaturated fatty acids | 28100.0 | 25900.0 | 29500.0 | | 27600.0 | 28600.0 | 25300.0 | 28700.0 | 27600.0 | 28300.0 |
| Total sum of fatty acids | 101000.0 | 74200.0 | 95400.0 | | 85600.0 | 93700.0 | 54900.0 | 86300.0 | 84800.0 | 95300.0 |
| The symbol "<" in the result column means that the substance concerned was not quantifiable as it was below the limit of detection. The sum calculations were done without taking into account values below the detection limit.  Control = Conventional corn-wheat based broiler diet  L-low = 5% of the daily dry matter feed intake is replaced by live black soldier fly larvae  MO-low = 5% of the daily dry matter feed intake is replaced by a mix of black soldier fly larvae meal & oil  M-low = the same amount of black soldier fly larvae meal is used as in diet MO-low  O-low = the same amount of black soldier fly larvae oil is used as in diet MO-low | | | | L-high = 10% of the daily dry matter feed intake is replaced by live black soldier fly larvae  MO-high = 10% of the daily dry matter feed intake is replaced by a mix of black soldier fly larvae meal & oil  M-high = the same amount of black soldier fly larvae meal is used as in diet MO-high  O-high = the same amount of black soldier fly larvae oil is used as in diet MO-high | | | | | | |

**Table S 15**: Temperature and humidity schedule for the experimental rooms during the 7 weeks of the trial

| Broiler age (d) | Temperature (°C) | Relative humidity (%) |  |
| --- | --- | --- | --- |
| Before arrival | 34 | 50-60 |  |
| 0-1 | 33 | 50-60 |  |
| 2 | 32 | 50-60 |  |
| 3 | 31 | 50-60 |  |
| 4 | 30 | 50-60 |  |
| 5 to 6 | 29 | 50-60 |  |
| 7 to 8 | 28 | 40-60 |  |
| 9 to 11 | 27 | 40-60 |  |
| 12 to 13 | 26 | 40-70 |  |
| 14 to 16 | 25 | 40-70 |  |
| 17 to 20 | 24 | 40-70 |  |
| 21 to 23 | 23 | 40-70 |  |
| 24 to 27 | 22 | 40-70 |  |
| 28 to 31 | 21 | 40-70 |  |
| 32 to 35 | 20 | 40-70 |  |
| 36 to 39 | 19 | 40-70 |  |
| 40 to 52 | 18 | 40-70 |  |

**Table S 16**: Light and dark schedule in the experimental rooms during the 7 weeks of the trial

| Broiler age (d) | Light:Dark schedule (h) |
| --- | --- |
| 0 to 1 | 24:0 |
| 2 to 3 | 22:2 |
| 4 to 7 | 20:4 |
| 8 to 52 | 18:6 |


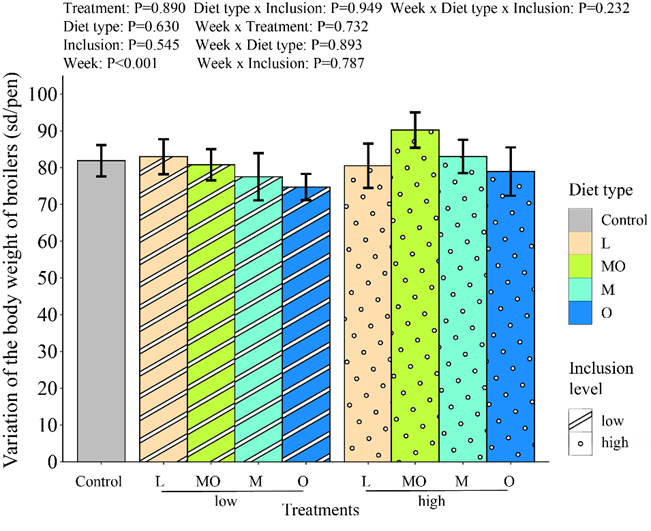


**Figure S 1**: Variation of the body weight of slow-growing broilers per pen during 7 weeks of growth. The individual BW of broilers within one pen was measured biweekly. Data is presented as mean standard deviation ± standard error. None of the measured factors and factor interactions were significant as indicated in the figure. Different bars indicate the different dietary treatments (Control = Conventional corn-wheat based broiler diet, L-low = 5% of the daily dry matter feed intake is replaced by live black soldier fly larvae, MO-low = 5% of the daily dry matter feed intake is replaced by a mix of black soldier fly larvae meal & oil, M-low = the same amount of black soldier fly larvae meal is used as in diet MO-low, O-low = the same amount of black soldier fly larvae oil is used as in diet MO-low, L-high = 10% of the daily dry matter feed intake is replaced by live black soldier fly larvae, MO-high = 10% of the daily dry matter feed intake is replaced by a mix of black soldier fly larvae meal & oil, M-high = the same amount of black soldier fly larvae meal is used as in diet MO-high, O-high = the same amount of black soldier fly larvae oil is used as in diet MO-high). Every treatment had eight replicates. The control group was excluded in the contrast of the interaction between diet type and inclusion.
